# Research hotspots and trends for Duchenne muscular dystrophy: a machine learning bibliometric analysis from 2004 to 2023

**DOI:** 10.3389/fimmu.2024.1429609

**Published:** 2024-11-28

**Authors:** Pingping Fang, Jingzhe Han, Di An, Yi Bu, Guang Ji, Mingjuan Liu, Jinliang Deng, Moran Guo, Xu Han, Hongran Wu, Shaojuan Ma, Xueqin Song

**Affiliations:** ^1^ Department of Neurology, Hebei Medical University, Shijiazhuang, Hebei, China; ^2^ Department of Neurology, Handan Central Hospital, Handan, Hebei, China; ^3^ Department of Neurology, Hengshui People’s Hospital, Hengshui, Hebei, China; ^4^ Department of Neurology, Affiliated Hospital of Hebei University, Baoding, Hebei, China; ^5^ Department of Neurology, Affiliated Hospital of Chengde Medical University, Chengde, Hebei, China; ^6^ Department of Neurology, The Second Hospital of Hebei Medical University, Shijiazhuang, Hebei, China; ^7^ Department of Neurology, Peking University First Hospital, Beijing, China; ^8^ Key Laboratory of Clinical Neurology (Hebei Medical University), Ministry of Education, Shijiazhuang, Hebei, China; ^9^ Neurological Laboratory of Hebei Province, Shijiazhuang, Hebei, China

**Keywords:** Duchenne muscular dystrophy, bibliometric, CiteSpace VOSviewer, knowledgemap, hotspots, topics

## Abstract

**Aims:**

The aim of this study was to conduct a bibliometric analysis of the relevant literature on Duchenne muscular dystrophy (DMD) to ascertain its current status, identify key areas of research and demonstrate the evolution of the field.

**Methods:**

The analysis sourced documents from the Science Citation Index Expanded in the Web of Science core collection, utilizing CiteSpace software and an online bibliometric platform to analyze collaborative networks among authors, institutions and countries, and to map out the research landscape through journal and reference evaluations. Keyword analyses, including clustering and emergent term identification, were conducted, alongside the development of knowledge maps.

**Results:**

The study included 9,277 documents, indicating a rising publication trend in the field. The Institut National de la Santé et de la Recherche Médicale emerged as the top publishing institution, with Francesco Muntoni as the most prolific author. The United States dominated in publication output, showcasing significant leadership. The keyword analysis highlighted 786 key emergent terms, primarily focusing on the mechanisms, diagnostics and treatment approaches in DMD.

**Conclusion:**

The field of DMD research is experiencing robust growth, drawing keen interest globally. A thorough analysis of current research and trends is essential for advancing knowledge and therapeutic strategies in this domain.

## 1 Introduction

Duchenne muscular dystrophy (DMD) is a severe, progressive, muscle-wasting disease (1). Its earliest symptoms are difficulties with climbing stairs, a waddling gait and frequent falls; patients present with these symptoms around 2–3 years of age (2). Most patients become wheelchair dependent around 10–12 years of age and need assisted ventilation at around 20 years of age (1). With optimal care, most patients with DMD die between 20 and 40 years of age from cardiac and/or respiratory failure (3).

DMD is caused by mutations in the DMD gene (encoding dystrophin) that prevent the production of the muscle isoform of dystrophin (Dp427m) (3). Mutations in the DMD gene can also cause Becker muscular dystrophy (4), which is a milder disease with a later onset and a slower progression than DMD. The different spectra of the diseases can be explained by the ‘reading frame rule’ (5). In muscle, dystrophin links cytoskeletal F-actin with the extracellular matrix via its N-terminal and C-terminal domains. In DMD, frameshifting mutations (deletions or duplications that involve a number of nucleotides not divisible by three) or nonsense mutations (a point mutation where the code for a codon for an amino acid is changed into a stop codon) cause premature truncation of protein translation leading to non-functional and unstable dystrophin. Nonsense-mediated decay does not seem to affect these dystrophin transcripts, but epigenetic changes cause a reduction in transcript production (6).

In recent years, several randomized controlled trials (7–10) and meta-analyses (11–14) have provided detailed insights into the clinical features, trends and therapeutic advancements in DMD. However, analyses that examine the research trends, depth, and focal points in DMD are lacking. Assessing the advancements in clinical application, current status, research hotspots and future directions of DMD diagnostic studies is of great importance for the broader clinical application and deeper foundational research of this field. Furthermore, the exponential growth of literature related to DMD has made it challenging to showcase the field’s full landscape and advancements through manual literature retrieval. Thus, a macroscopic redefinition of this topic within the published literature is necessary.

The field of bibliometrics was defined by Pritchard in 1969 as a discipline that employs mathematical and statistical methods to analyze literature, books and other forms of community knowledge. This technique, which has been widely applied across various scientific domains (15), serves as an effective tool for literature analysis. Bibliometrics is an interdisciplinary field that quantitatively analyses knowledge carriers using mathematical and statistical approaches. The scientific knowledge map, which is a visual representation of scientific knowledge, allows for the exploration, drawing, analysis, summarization and revelation of knowledge structures and domains across temporal and spatial dimensions (16). In the last few years, the integration of knowledge visualization with bibliometrics has facilitated the intuitive and vivid representation of information, including research patterns, structural relationships and developmental processes of knowledge (17, 18). Among the plethora of visualization analysis software, such as Sci2, CiteSpace and VOS viewer, CiteSpace, developed in Java by Professor Chen CM, is one of the most popular tools for crafting knowledge graphs (19). It is renowned for supporting multiple data formats, comprehensive functionalities and effective visualization effects, and has been employed in numerous disciplinary studies (20). As a developing field, bibliometrics has been extensively used in various medical fields, including internal medicine and surgery/pediatrics (21–23). Despite the recent exponential increase in bibliometric literature, visual quantitative analysis of DMD research remains lacking. Literature from the past two decades holds significant referential value for understanding the current research landscape and guiding future research directions. Consequently, this study employed bibliometric methods and scientific knowledge maps to utilize CiteSpace software for a visual analysis of the international progress and current status of DMD research over the past two decades from various perspectives. such as document distribution and co-occurrence maps. In addition, it unveiled the research hotspots and future trends through timeline views and burst word trajectories, offering insights into research frontiers. Moreover, this study curated a selection of scientific literature on DMD to provide references and data support for international DMD research.

## 2 Materials and methods

### 2.1 Data source

**Figure 1:**
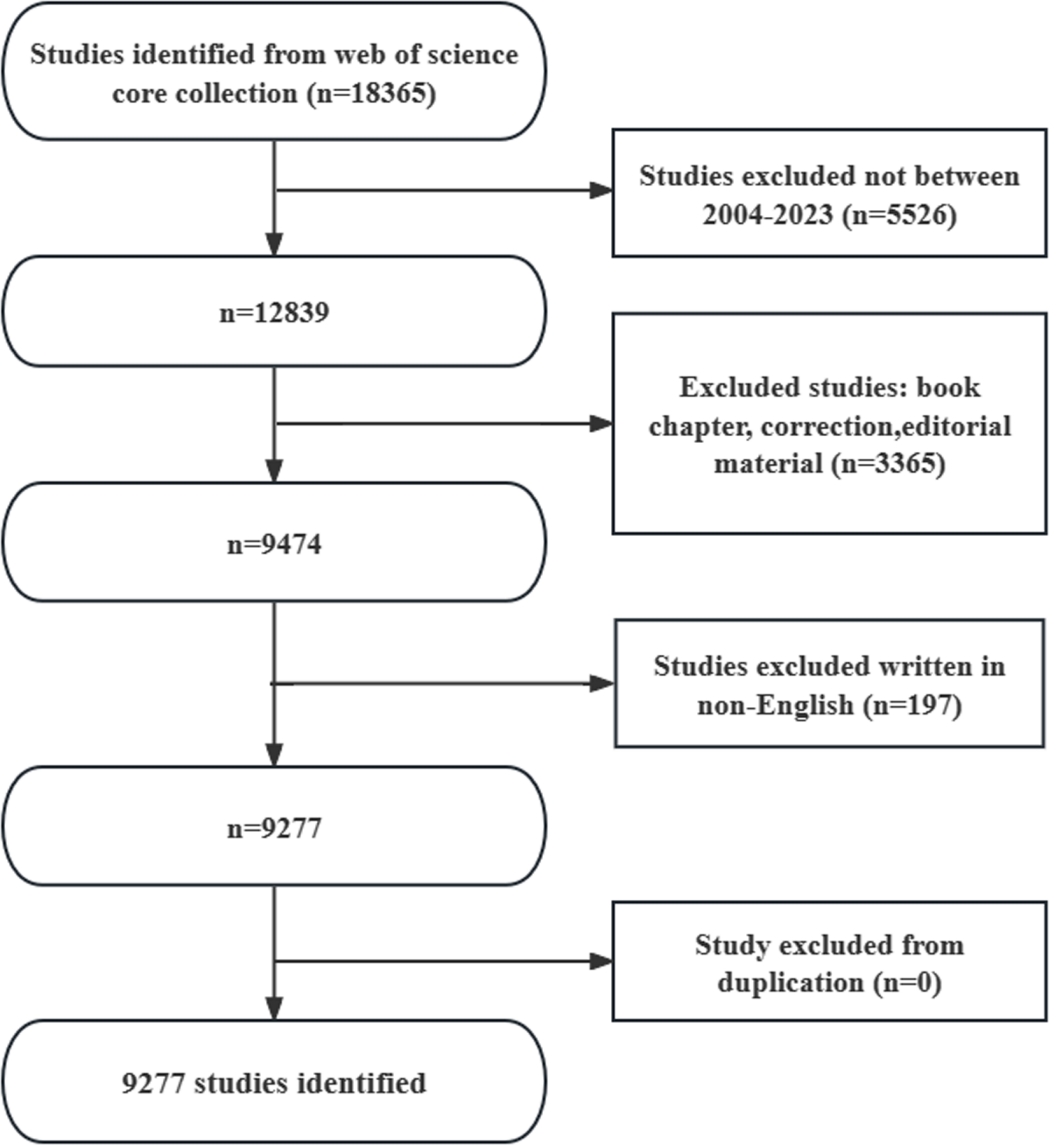
Literature Search Flowchart (Created using CiteSpace version: 6.1.6R on January 17, 2024. Also utilized VOSviewer version: 1.6.17).

Based on the Web of Science Core Collection (WoSCC), our search on 17 January 2024 retrieved articles spanning from 1 January 2004 to 31 December 2023 focused on various terminologies associated with DMD. The extensive search string incorporated a range of terms to ensure comprehensive coverage: TS=(Muscular Dystrophy, Duchenne or Cardiomyopathy, Dilated, X-Linked OR Childhood Muscular Dystrophy, Pseudohypertrophic or Childhood Pseudohypertrophic Muscular Dystrophy or Duchenne Muscular Dystrophy or Duchenne-Type Progressive Muscular Dystrophy or Duchenne Type Progressive Muscular Dystrophy or Muscular Dystrophy, Childhood, Pseudohypertrophic or Muscular Dystrophy, Duchenne Type or Muscular Dystrophy, Pseudohypertrophic or Pseudohypertrophic Muscular Dystrophy or Muscular Dystrophy, Pseudohypertrophic Progressive, Duchenne Type or Muscular Dystrophy, Pseudohypertrophic, Childhood or Progressive Muscular Dystrophy, Duchenne Type or Pseudohypertrophic Childhood Muscular Dystrophy or Pseudohypertrophic Muscular Dystrophy, Childhood or Cardiomyopathy, Dilated, 3B or Duchenne and Becker Muscular Dystrophy or Muscular Dystrophy, Duchenne and Becker Types or Duchenne-Becker Muscular Dystrophy or Duchenne Becker Muscular Dystrophy or Muscular Dystrophy, Duchenne-Becker or Becker Muscular Dystrophy or Muscular Dystrophy, Pseudohypertrophic Progressive, Becker Type or Muscular Dystrophy, Becker or Muscular Dystrophy, Becker Type or Becker’s Muscular Dystrophy or Muscular Dystrophy, Becker’s or Muscular Dystrophy Pseudohypertrophic Progressive, Becker Type) (**Figure 1**).

### 2.2 Data establishment and processing

Eligibility criteria for literature screening were established as follows (1): full-text publications pertinent to DMD (2); articles and review manuscripts written in English; and (3) publications dated between 1 January 2004 and 31 December 2023. Exclusion criteria included the following (1): topics unrelated to DMD (2); original articles formatted as conference abstracts, news briefs, etc. Plain text versions of the articles were exported for further analysis.

Graphpad Prism v8.0.2 was utilized to analyze and visualize the trends and proportions of publications by year and by country. CiteSpace (6.2.4R 64-bit advanced edition) and VOSviewer (1.6.18 version) were employed to further dissect the data and facilitate the creation of scientific knowledge maps. VOSviewer v.1.6.17, developed by Waltman et al. in 2009, is a Java-based software that offers free services for large-scale bibliographic data analysis and displays them in a map format. Professor Chaomei Chen’s CiteSpace (6.1.6R) software, which envisions an experimental framework for exploring new concepts and evaluating existing technologies, allows for an enriched understanding of knowledge domains, research frontiers and trends, as well as the prediction of future research trajectories.

## 3 Results

### 3.1 Number of literature and general characteristics

**Figure 2:**
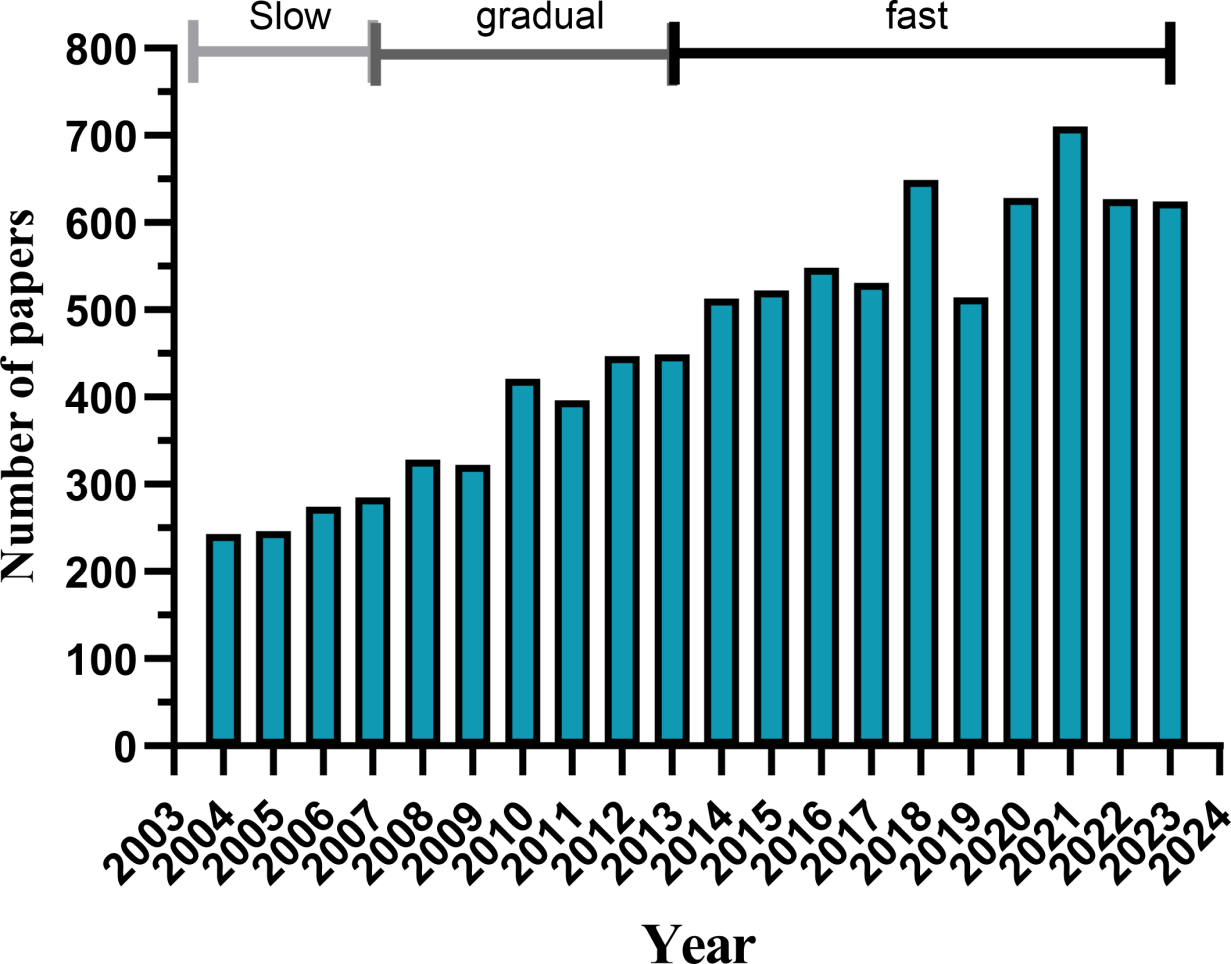
Annual Publication Volume (Created using Cite Space version: 6.1.6R on January 17, 2024. Also utilized VOSviewer version: 1.6.17.).

From 1 January 2004 to 31 December 2023, a total of 9,277 publications on DMD exist in the WoSCC database, including 7,744 articles (84.96%) and 1,533 reviews (15.04%). These publications involved 104 countries and regions, 5,622 institutions and 32,992 authors. Since 2004, the number of papers published annually has slowly increased. We divide this increase into three phases. From 2004 to 2007, the growth was slow (**Figure 2**), with fewer than 300 papers published annually, indicating that the field was not a focus of researchers. From 2008 to 2013, the number of publications gradually increased, showing that the field was gaining researchers’ attention. After 2014, the publication volume in the field increased rapidly and reached a peak in 2021, indicating widespread attention to this field after 2014.

### 3.2 National and institutional affiliation analysis

**Figure 3:**
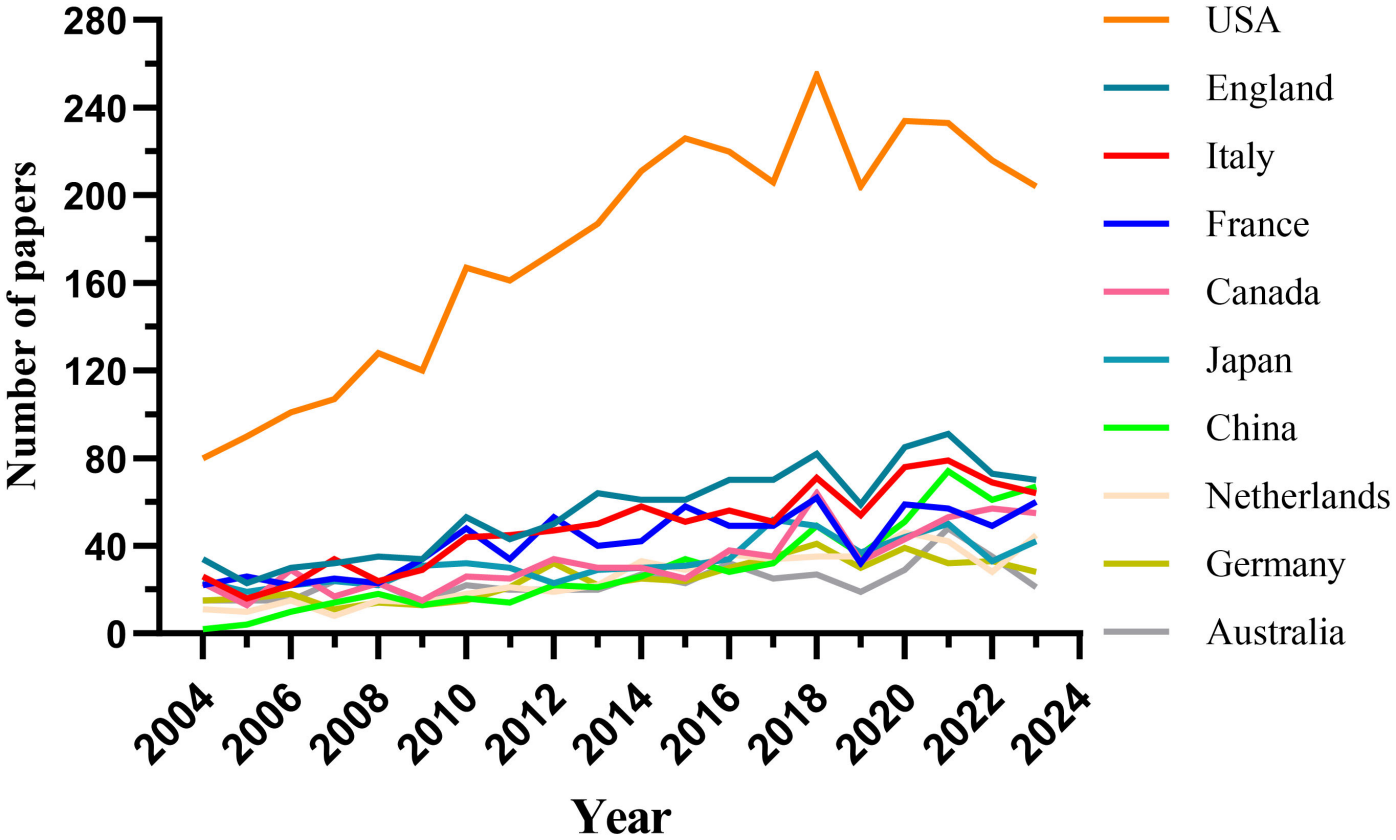
National Publication Volume Line Chart (Created using CiteSpace version: 6.1.6R on January 17, 2024. Also utilized VOSviewer version: 1.6.17.).

**Figure 4:**
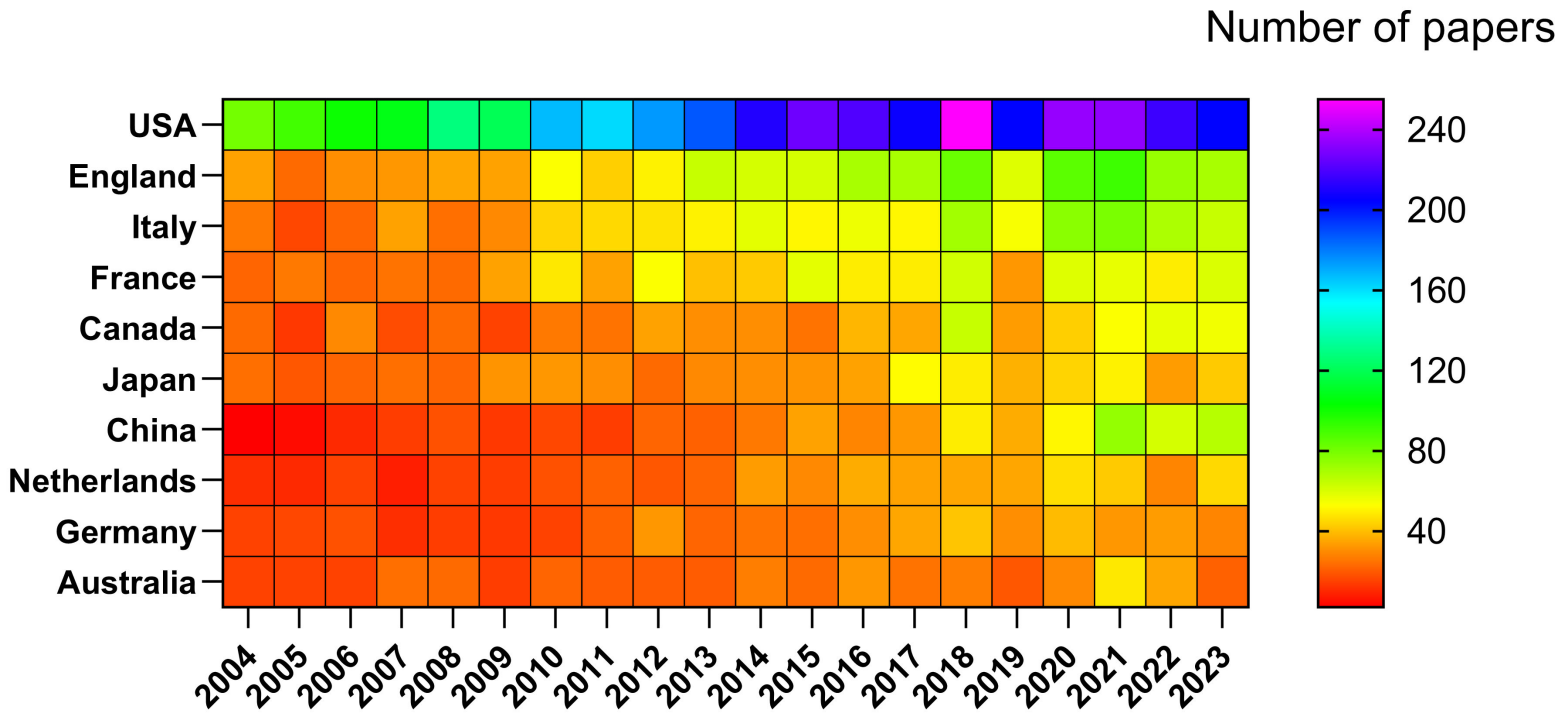
Heat Map of National Publication Volumes (Created using CiteSpace version: 6.1.6R on January 17, 2024. Also utilized VOSviewer version: 1.6.17.).

Research pertaining to DMD has been conducted across 104 countries and regions. The annual publication outputs of the top 10 contributing countries over the previous decade are presented in **Figures 3** and **4**. The leading nations in this research domain are the USA, the UK, Italy, France and Canada. Notably, publications from the USA constitute 37.99% of the total output, markedly exceeding that of any other country (**Figure 4**, **Supplementary Table 1**).

**Figure 5:**
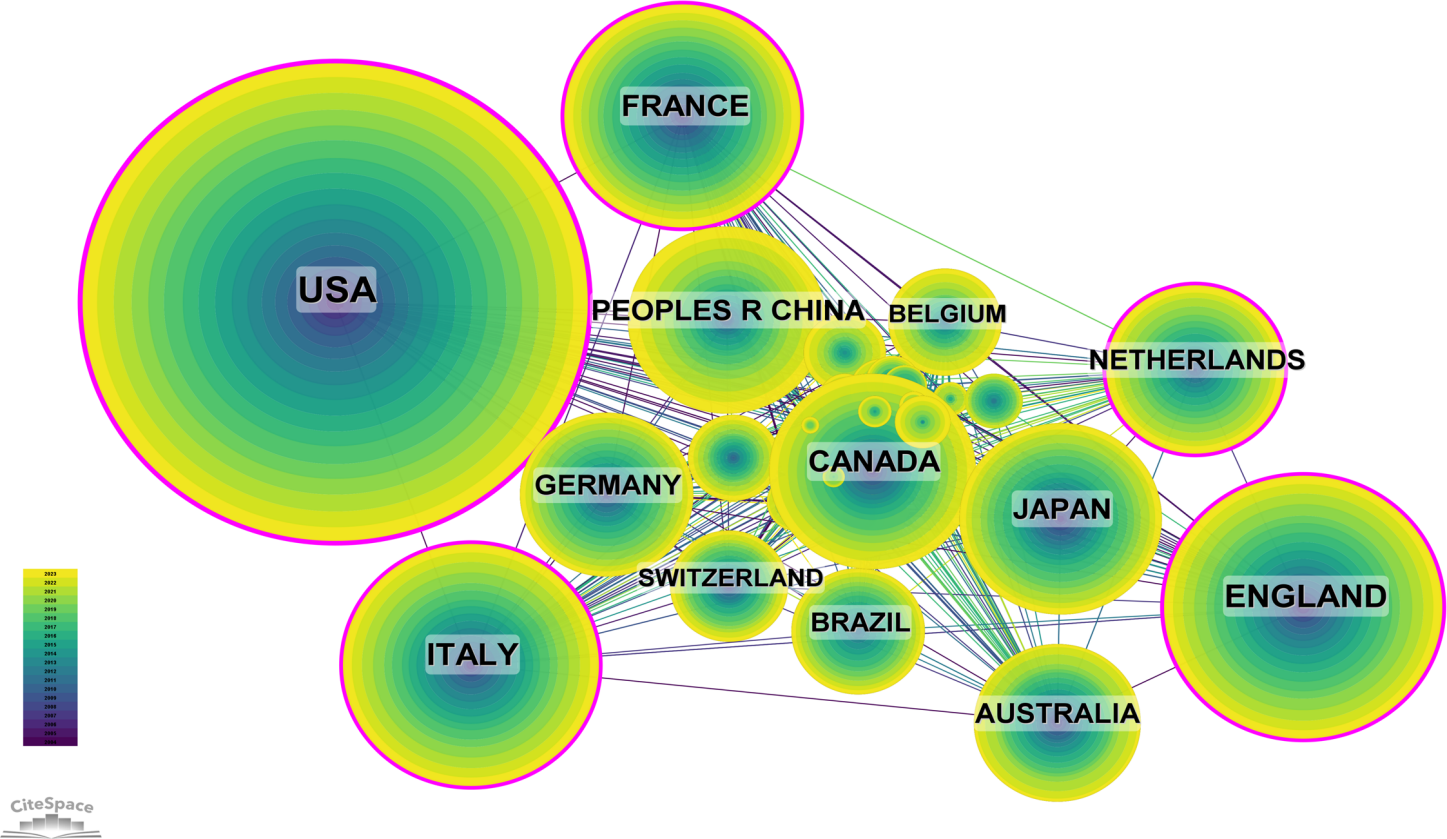
Map of International Collaboration Network Among Countries (Created using CiteSpace version: 6.1.6R on January 17, 2024. Also utilized VOSviewer version: 1.6.17.).

Among the top 10 countries/regions by publication output, the USA’ research papers were cited 153,524 times (**Supplementary Table 1**), markedly exceeding the citation figures of other nations. Its citation-to-publication ratio of 43.57 is the highest among all countries/regions, reflecting the high quality of its research outputs. The UK ranked second in publication volume with 1,120 papers and has a citation count of 45,035, thus having the second-highest citation-to-publication ratio of 40.21. **Figure 5** depicts the collaborative networks, highlighting intensive collaborations between the highest producing countries, namely, the USA and the UK. The USA maintains robust research collaborations with France, China and Italy, whereas the UK has closer research ties with Japan, Australia and the Netherlands. The centrality index of the USA is 0.27, indicating its pivotal role in this research field. The significant increase in publication volumes in countries such as the UK and France in recent years may be attributed to their collaborations with the USA.

**Figure 6:**
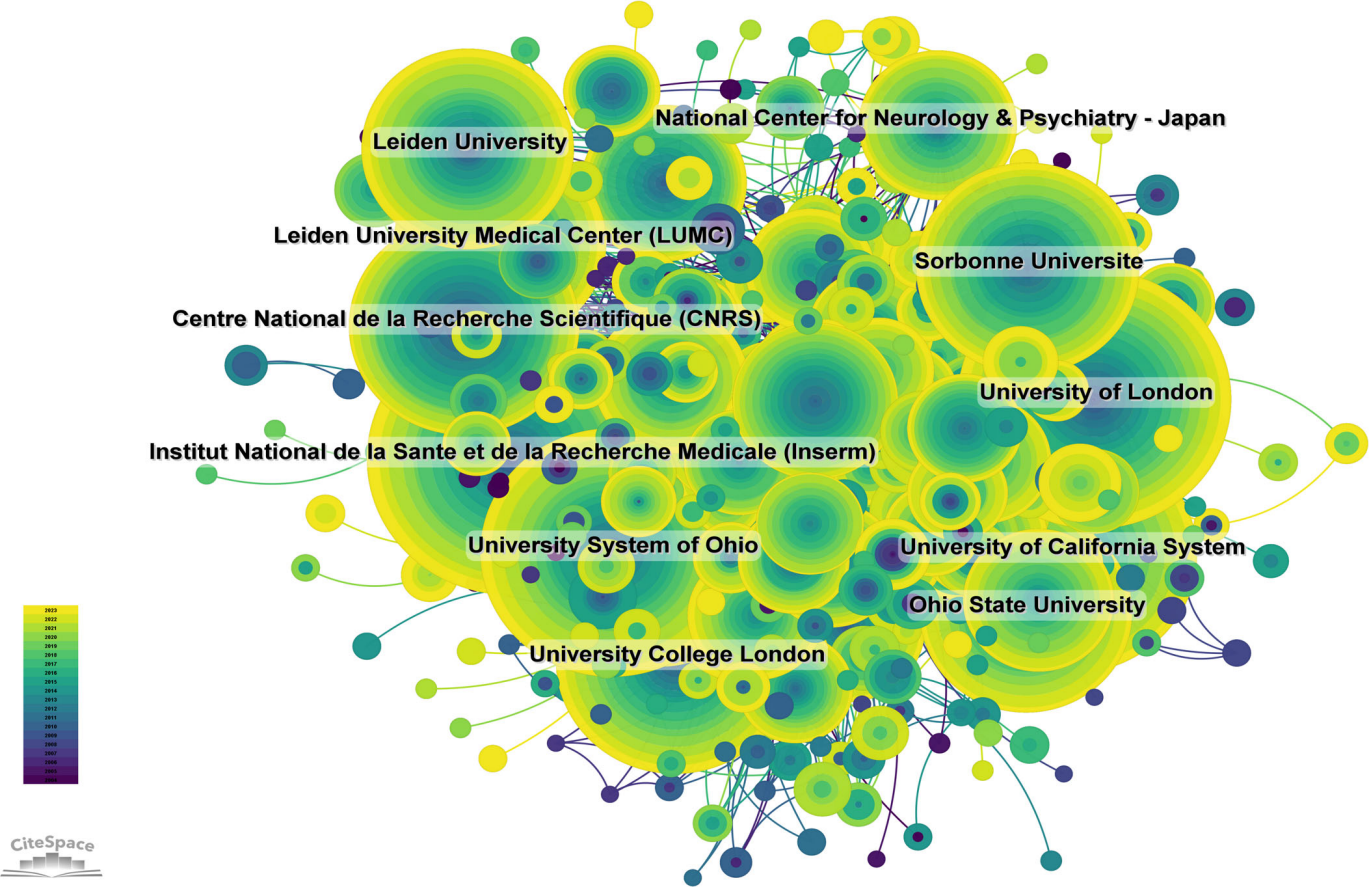
Institutional Collaboration Network Map (Created using CiteSpace version: 6.1.6R on January 17, 2024. Also utilized VOSviewer version: 1.6.17.).

A total of 5,622 institutions systematically published articles on DMD. Among the top 10 institutions by publication volume, three are from the USA, three are from France, two are from the UK and two are from the Netherlands (**Supplementary Table 2**, **Figure 6**). The French National Institute of Health and Medical Research (Inserm) published the most papers (483 papers with 17,546 citations, averaging 36.33 citations per paper). The University of London ranked second with 451 papers and 18,002 citations, resulting in an average of 39.92 citations per paper. The University of California System ranked third with 423 papers and 23,662 citations, averaging 55.94 citations per paper. Further analysis revealed a tendency among domestic institutions to collaborate more frequently with other institutions within their own country. Thus, we call for enhanced collaboration between domestic and international institutions to break down academic barriers.

### 3.3 Journal analysis

**Figure 7:**
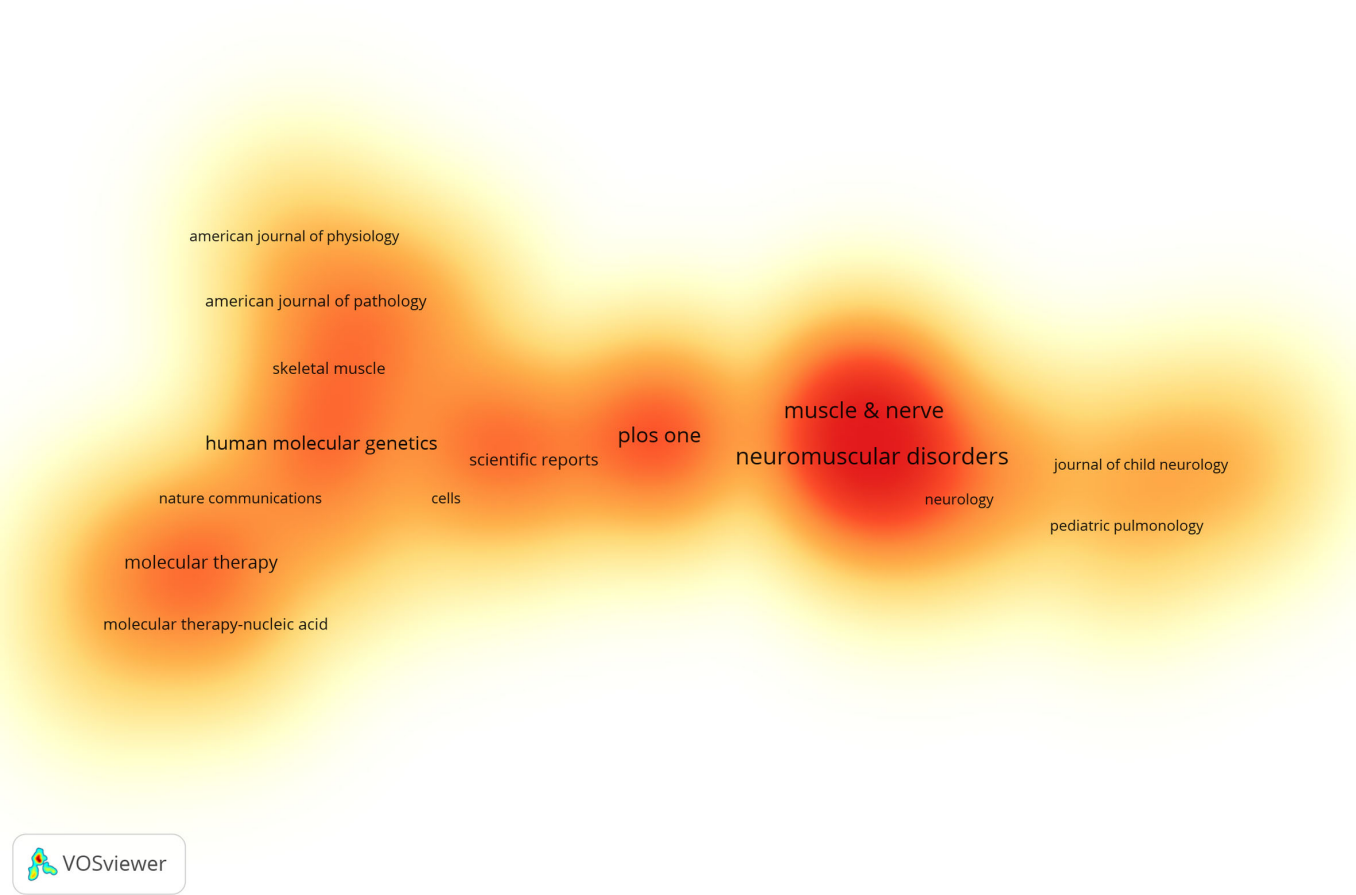
Journal Publication Density Map (Created using CiteSpace version: 6.1.6R on January 17, 2024. Also utilized VOSviewer version: 1.6.17.).

The journals with the highest publication volume and the most citations are listed in **Supplementary Tables 3** and **4**. *Neuromuscular Disorders* is the most prolific journal in the field, publishing 396 articles, which represent 4.27% of the total publications in this domain. It is followed by *Muscle & Nerve* with 326 articles (3.51%), *PLOS One* with 298 articles (3.21%) and *Human Molecular Genetics* with 182 articles (1.96%). Among the top 10 most productive journals, *Molecular Therapy* boasts the highest impact factor (IF) of 12.4. In addition, 80% of these journals are ranked within the first or second quartiles (Q1 or Q2), indicating their high standing within the scientific community (**Figure 7**).

**Figure 8:**
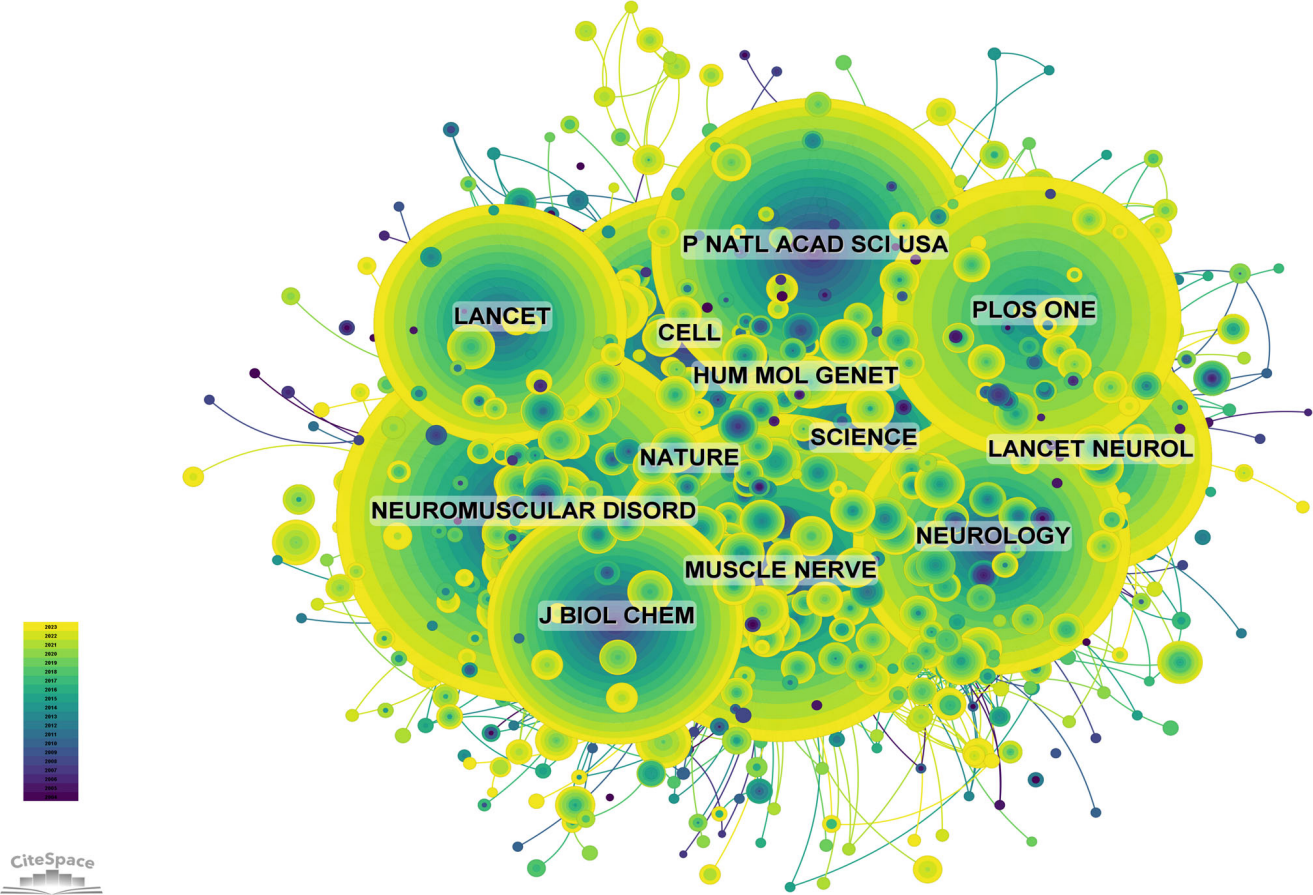
Co-citation Network Map of Journals (Created using CiteSpace version: 6.1.6R on January 17, 2024. Also utilized VOSviewer version: 1.6.17.).

The impact of a journal is determined by its frequency of co-citations, which indicates the significant influence of the journal on the scientific community. **Figure 8** and **Supplementary Table 4** provide a graphical representation of the co-citation frequencies, demonstrating that Neuromuscular Disorders holds a prominent position in the literature with a total of 5,769 citations, followed by Muscle & Nerve (4,892 citations) and Proceedings of the National Academy of Sciences of the United States of America (4,282 citations). Among the top 10 journals by co-citation count, *Nature* is cited 3,971 times and has the highest IF at 64.8 among these journals. In addition, 80% of the journals in the co-citation analysis are ranked in the Q1/Q2 quartiles.

**Figure 9:**
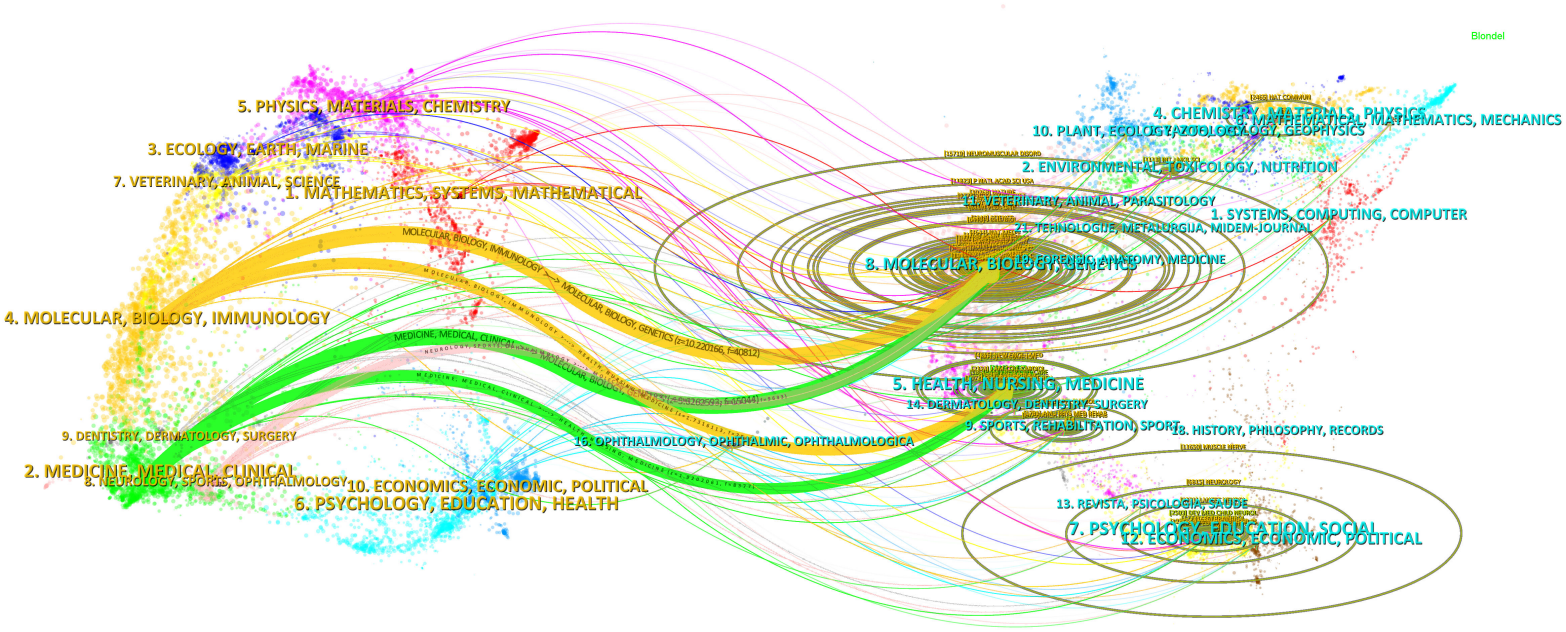
Journal Overlay Map (Created using CiteSpace version: 6.1.6R on January 17, 2024. Also utilized VOSviewer version: 1.6.17.).

The topic distribution of academic publications over the last 20 years is illustrated using a double map overlay. The colored trajectories represent citation links, with citing journals on the left and cited journals on the right. On the basis of the results, we have identified four major citation pathways. Research published in journals from the molecular/biology/immunology domain is primarily cited by research from the molecular/biology/genetics and health/nursing/medicine domains. Conversely, research from the medicine/medical/clinical and health/nursing/medicine domains is predominantly cited by publications in the molecular/biology/genetics domain (**Figure 9**).

### 3.4 Author and co-cited authors

**Figure 10:**
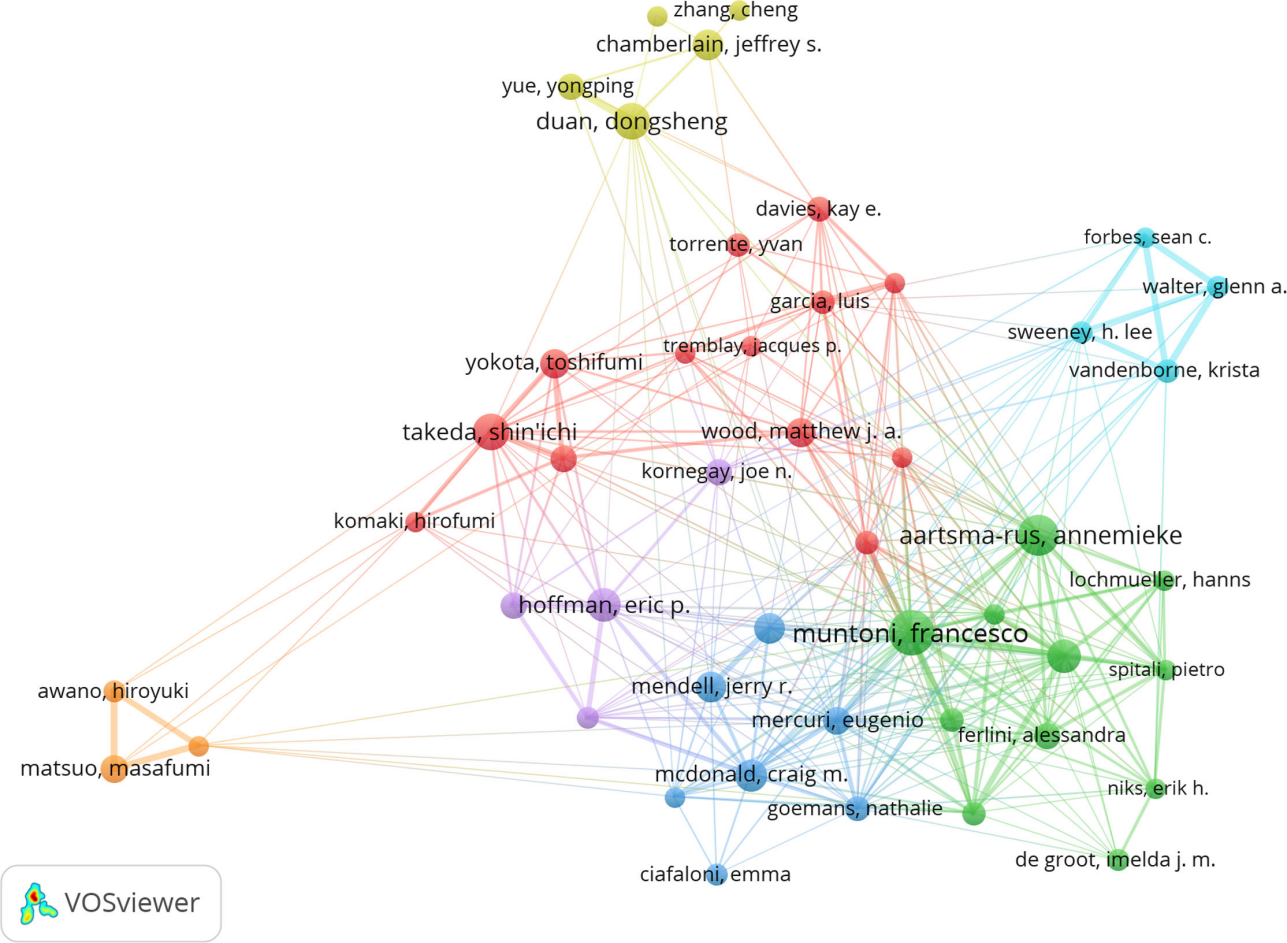
Author Collaboration Network Map (Created using CiteSpace version: 6.1.6R on January 17, 2024. Also utilized VOSviewer version: 1.6.17.).

In the last 20 years, the top 10 authors in terms of number of publications in the field of DMD research have published 913 papers, representing 9.84% of the total academic output (**Supplementary Table 5**). Francesco Muntoni leads with 140 publications, followed by Annemieke Aartsma-Rus with 118 and Shin’ichi Takeda with 99. Of the top 10 most prolific authors, eight are based in the USA, one is in the Netherlands and one is based in China. The network of collaborations among these authors is visualized using CiteSpace (**Figure 10**).

**Figure 11:**
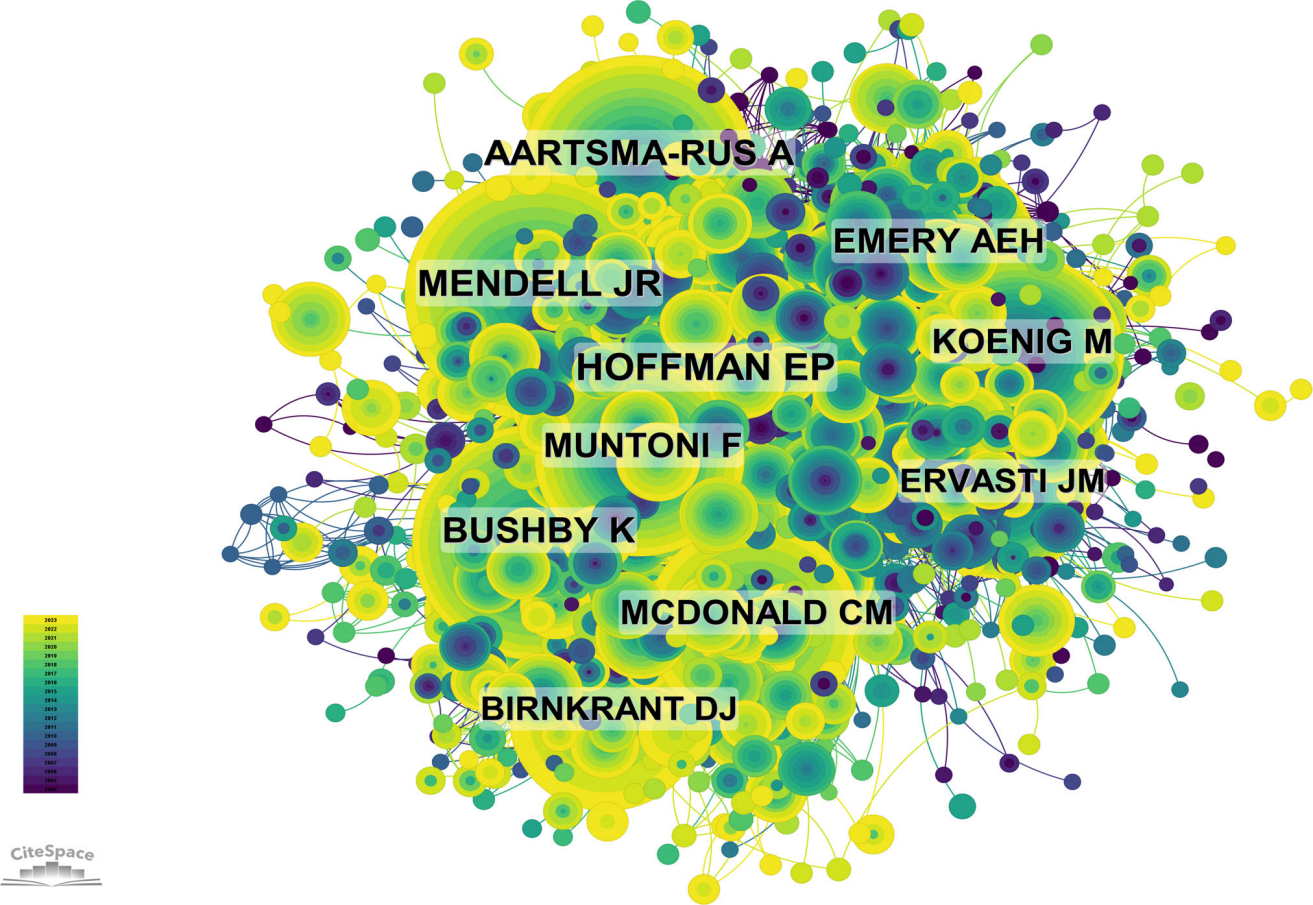
Co-citation Network Map of Authors. (Created using CiteSpace version: 6.1.6R on January 17, 2024. Also utilized VOSviewer version: 1.6.17.).

This study identifies the top 10 authors with the highest co-citation and citation counts in DMD research over the past 20 years, as illustrated in **Figure 11** and summarized in **Supplementary Table 5**.A total of 132 authors have been cited more than 50 times, indicating their research is highly reputable and influential. The largest node is associated with the most frequently co-cited authors, including Hoffman E. P. (cited 2,099 times), Mendell J.R. (cited 1,531 times), and Bushby K. (cited 1,402 times). Further investigation reveals that Hoffman ranks fourth in terms of publication volume and first in citation count, highlighting his leadership in the field (**Figures 11**).

**Figure 12:**
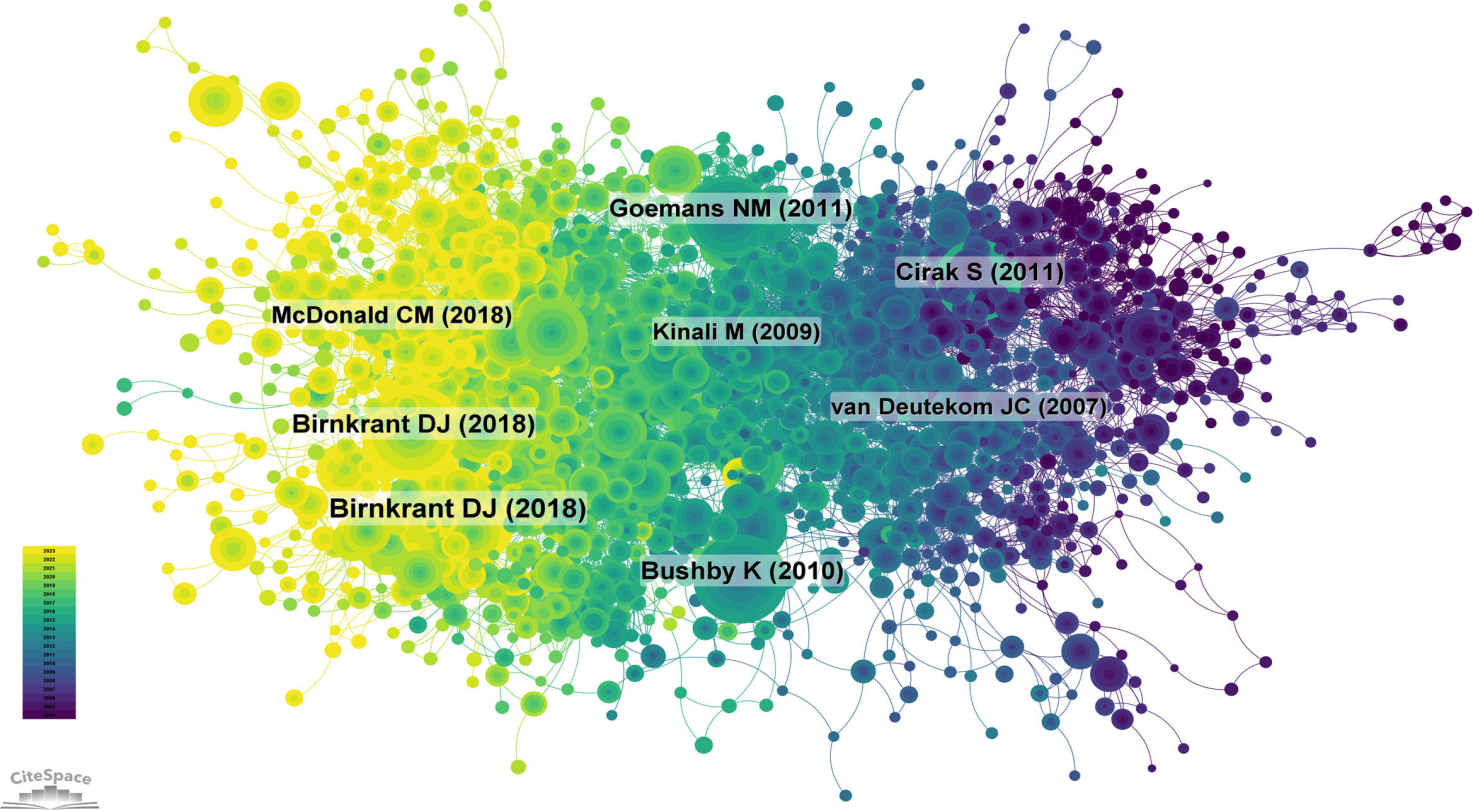
Co-citation Network Map of References. (Created using CiteSpace version: 6.1.6R on January 17, 2024. Also utilized VOSviewer version: 1.6.17.).

From 2004 to 2023, the co-cited reference network contains 2,060 nodes and 11,338 links (**Figure 12**). Among the top 10 most frequently co-cited articles (**Supplementary Table 6**), the article titled ‘Diagnosis and management of Duchenne muscular dystrophy, part 1: diagnosis, and neuromuscular, rehabilitation, endocrine, and gastrointestinal and nutritional management’ published in *Lancet Neurology* (IF = 48.00) is the most frequently co-cited reference. Birnkrant is the first author of this paper. Since the publication of the Duchenne muscular dystrophy (DMD) care considerations’ in 2010, multidisciplinary care for this severe progressive neuromuscular disorder has continued to evolve. With improvements in patient survival rates, diagnostic and treatment strategies have shifted towards more predictive approaches, with a renewed focus on patient quality of life. In 2014, a steering committee of interdisciplinary experts was formed to update the 2010 DMD care considerations to enhance patient care. The new care guidelines aim to meet the needs of patients with extended survival periods, provide guidance for the progress of assessments and interventions, and consider the impact of emerging DMD genetic and molecular therapies. The committee identified 11 topics to be included in the updated version, of which eight were already addressed in the initial care considerations. The three new topics include primary care and emergency management, endocrine management and care transitions throughout the life span. This update is divided into three parts. The first part introduces care considerations for DMD diagnosis, neuromuscular, rehabilitation, endocrine (growth, puberty and adrenal insufficiency) and gastrointestinal (including nutrition and swallowing difficulties) management.

The second most cited article, also authored by Birnkrant, published in *Lancet Neurology* and titled ‘Diagnosis and management of Duchenne muscular dystrophy, part 2: diagnosis, and pharmacological and psychosocial management’, argues that a coordinated multidisciplinary treatment approach is crucial for optimal management of primary and secondary complications of DMD. Enhanced diagnostic techniques and earlier therapeutic interventions have profoundly influenced contemporary care, potentially extending patient survival and improving quality of life. The second part of this updated DMD care considerations introduces the latest recommendations for the respiratory, cardiac, bone health and osteoporosis, and orthopedic and surgical treatment of male DMD patients. Guidance is also provided for the cardiac treatment of female carriers of pathogenic mutations. The new care guidelines acknowledge the impact of long-term corticosteroid use on the natural history of DMD and the need for care guidance throughout the lifespan as patient longevity increases. With the advent of new genetic and molecular therapies, the therapeutic landscape for DMD is undergoing significant changes.

The third most cited article by Bushby, also published in *Lancet Neurology* and titled ‘Diagnosis and management of Duchenne muscular dystrophy, part 1: diagnosis, and pharmacological and psychosocial management’, describes DMD as a severe progressive disease affecting one in 3,600–6,000 live male births. Although guidelines exist for various aspects of DMD, comprehensive clinical care advice was lacking. The Centers for Disease Control and Prevention selected 84 clinicians using the RAND Corporation-University of California, Los Angeles appropriateness method to establish care guidelines. The DMD Care Considerations Working Group assessed the evaluation and intervention measures for managing DMD diagnosis, gastroenterology and nutrition, rehabilitation, neuromuscular, psychosocial, cardiovascular, respiratory, orthopedic and surgical aspects. These guidelines are divided into two parts, intended for the wide range of professionals involved in caring for DMD patients, and provide a framework for understanding the multiple primary manifestations and secondary complications of DMD and delivering coordinated multidisciplinary care. In the first part of this review, we introduce the methods used to propose these recommendations, as well as the overall approach to care, pharmacological treatment and psychosocial management.

These three articles further emphasize that treatment for DMD is multidisciplinary, with nursing playing a significant role. These articles also represent foundational literature for the management of DMD.

**Figure 13:**
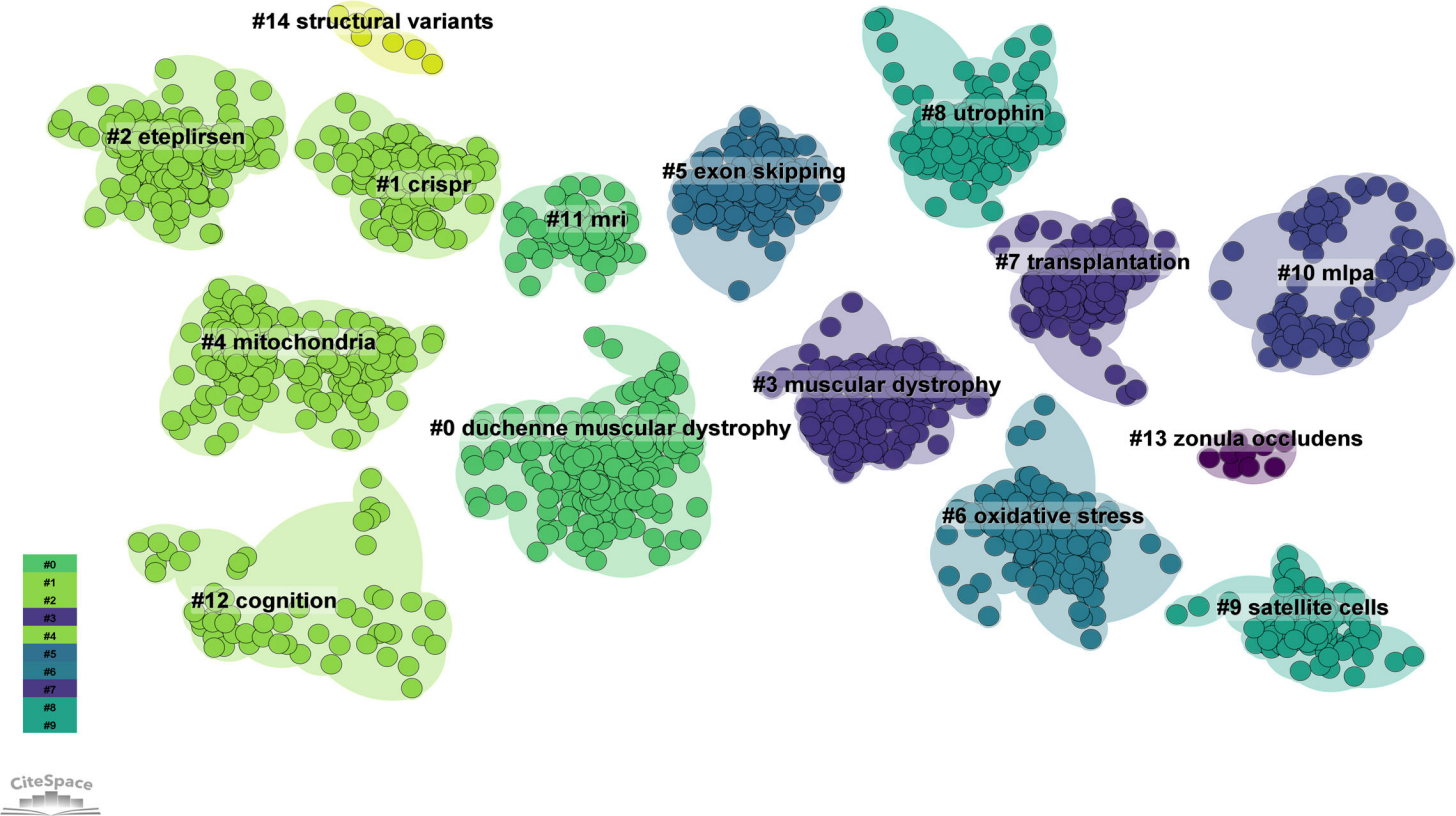
Cluster Map of Co-cited References. (Created using CiteSpace version: 6.1.6R on January 17, 2024. Also utilized VOSviewer version: 1.6.17.).

**Figure 14:**
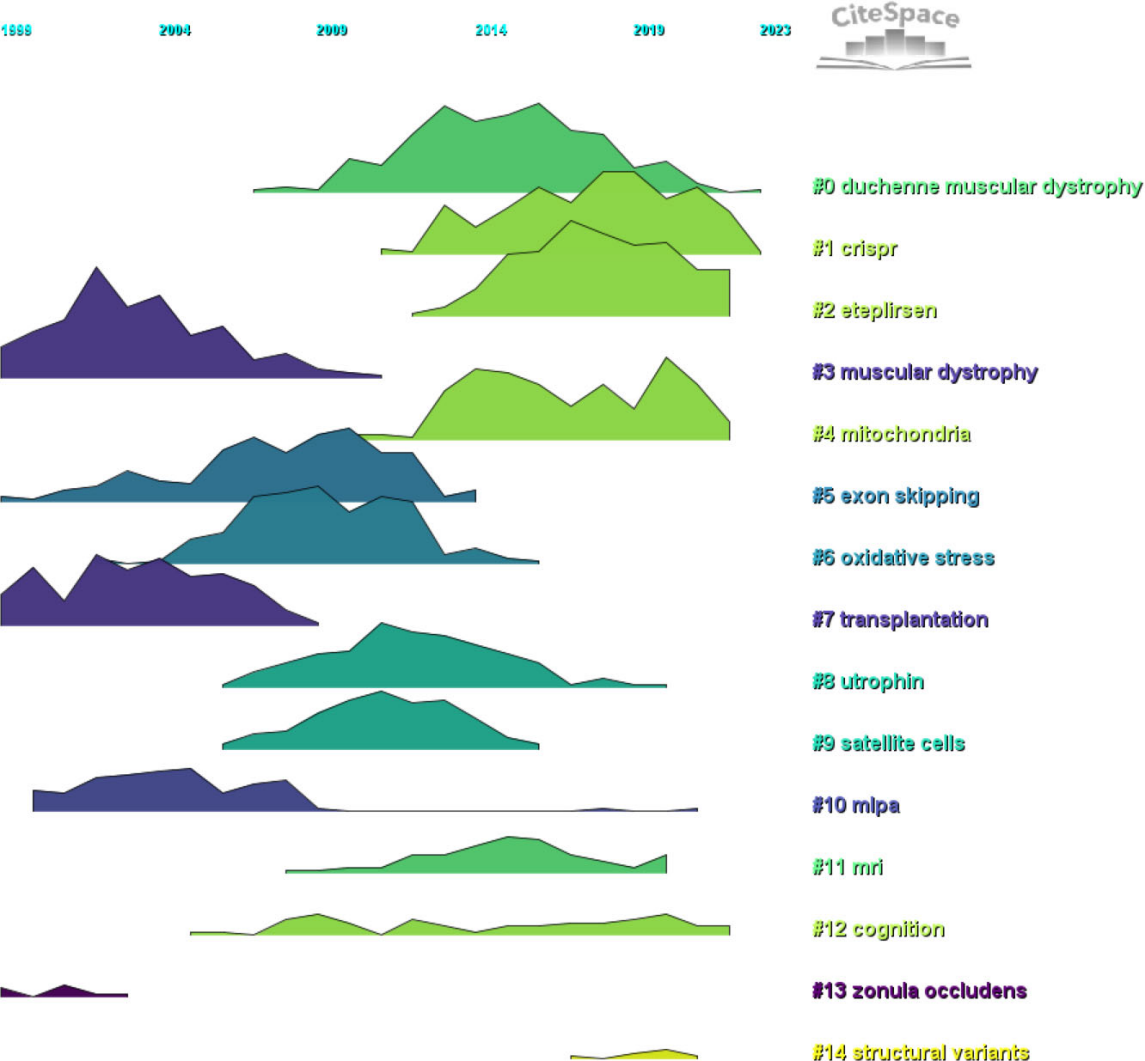
Volcano Plot of Co-cited References. (Created using CiteSpace version: 6.1.6R on January 17, 2024. Also utilized VOSviewer version: 1.6.17.).

We conducted co-citation reference clustering and temporal clustering analyses (**Figures 13** and **14**). We found that early research hotspots included muscular dystrophy (cluster 3), transplantation (cluster 7), MLPA (cluster 10) and zonula occludens (cluster 13). Mid-term research hotspots were exon skipping (cluster 5), oxidative stress (cluster 6), utrophin (cluster 8) and satellite cells (cluster 9). Current hot topics and trends in the field are DMD (cluster 0), CRISPR (cluster 1), eteplirsen (cluster 2), mitochondria (cluster 4), MRI (cluster 11), cognition (cluster 12) and structural variants (cluster 14). These clusters illustrate the evolution of research focus and emerging areas of interest within the field over time.

### 3.5 Keyword analysis

**Figure 15:**
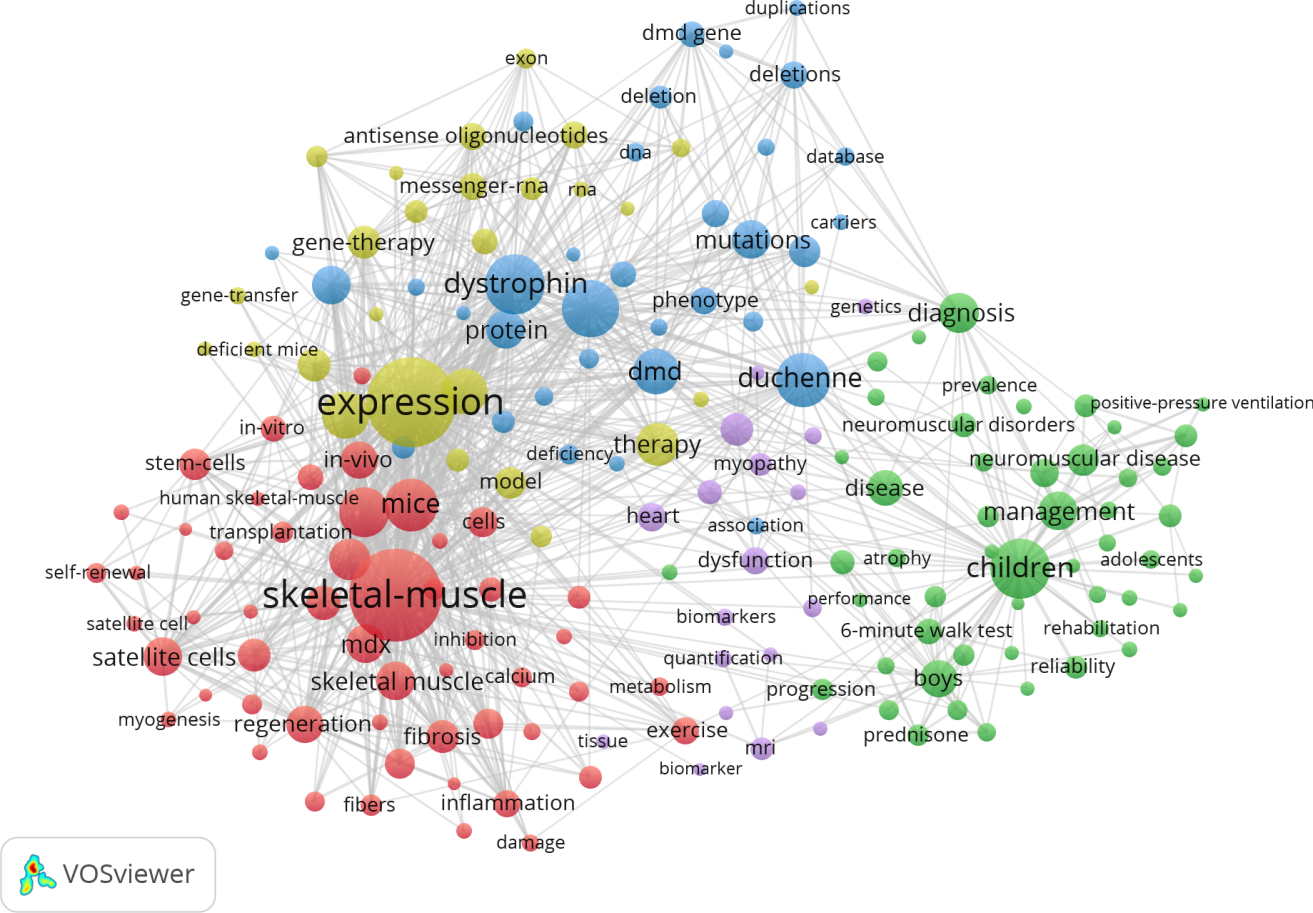
High-frequency Keyword Network Diagram. (Created using CiteSpace version: 6.1.6R on January 17, 2024. Also utilized VOSviewer version: 1.6.17.).

By analyzing keywords, we can quickly grasp the status and development direction of a field. On the basis of the co-occurrence of keywords in VOSviewer, the most popular keywords are ‘inflammation’ (269 occurrences), followed by ‘expression’ (207), ‘oxidative stress’ (143) and ‘activation’ (137) (**Supplementary Table 7**, **Figures 15** and **Supplementary Figure 1**). We removed irrelevant keywords and constructed a network of 180 keywords that appear at least 12 times, resulting in five distinct clusters. The first group (red) contains 53 keywords, including ‘skeletal-muscle’, ‘mdx’, ‘fibrosis’, ‘injury’, ‘differentiation’, ‘mice’, ‘stem-cell’, ‘self-renewal’, ‘regeneration’, ‘activation’, ‘damage’, ‘inflammation’, ‘proliferation’, ‘myogenesis’, ‘mechanisms’, ‘mitochondria’, ‘exercise’, ‘metabolism’, ‘growth’, ‘calcium’ and ‘animal models’. The second group (green) contains 51 keywords, including ‘children’, ‘boys’, ‘management’, ‘disease’, ‘atrophy’, ‘double-blind’, ‘disorders’, ‘prevalence’, adolescents’, ‘scoliosis’, ‘disability’, ‘health’, ‘reliability’, ‘neuromuscular disease’, ‘trial’, ‘deflazacort’, ‘natural history’ and ‘pulmonary function’. The third group (blue) includes 34 keywords, such as ‘gene’, ‘duchenne’, ‘dmd’, ‘dystrophin’, ‘protein’, ‘mutation’, ‘carriers’, ‘DNA’, ‘deletion’, ‘locus’, ‘duplications’, ‘phenotype’, ‘complex’, ‘cancer’, ‘deficiency’, ‘molecular basis’, ‘central nervous system’, ‘database’ and ‘association’. The fourth group (yellow) comprises 27 keywords, including ‘expression’, ‘mdx mouse’, ‘utrophin’, ‘gene therapy’, ‘delivery’, messenger RNA’, ‘restoration’, ‘systemic delivery’, ‘exon skipping’, ‘skeletal’, ‘canine model’, ‘pathology’ and ‘efficacy’. The fifth group (purple) contains 18 keywords, including ‘biomarker’, ‘age’, ‘disease progression’, ‘dysfunction’, ‘heart’, ‘heart failure’, ‘MRI’, ‘involvement’, ‘myopathy’ and ‘tissue’. We used CiteSpace to create a volcano plot to visually display the changes in research hotspots over time (**Supplementary Figures 2**, **3**). Please translate following the conventions of bibliometrics.

### 3.6 Co-cited references and keywords

Using CiteSpace, we identified 50 of the most reliable citation bursts in the field of DMD. The most frequently cited reference, with a citation rate of 148.96, is ‘Diagnosis and management of Duchenne muscular dystrophy, part 1: diagnosis, and neuromuscular, rehabilitation, endocrine, and gastrointestinal and nutritional management’, published in *Lancet Neurology*, with Birnkrant as the lead author. The article discusses the continuous development of multidisciplinary care for this severe progressive neuromuscular disease since the release of the DMD care considerations in 2010. As patient survival rates have increased, diagnostic and treatment strategies have shifted towards more proactive measures, with a renewed focus on improving patients’ quality of life. In 2014, a steering committee of experts from various disciplines was established to update the 2010 DMD care considerations to enhance patient care. The new guidelines aim to meet the needs of patients with extended life expectancy, guide the progression of assessments and interventions, and consider the impact of emerging DMD genetic and molecular therapies. The committee identified 11 topics to include in the updated guidelines, with eight previously discussed in the initial guidelines. Three new topics were primary care and emergency management, endocrine management and care transitions throughout the life span. The update is divided into three parts. The first part addresses the care considerations for DMD diagnosis, neuromuscular, rehabilitation, endocrine (growth, puberty and adrenal insufficiency) and gastrointestinal (including nutrition and swallowing difficulties) management. Out of the 50 references, 47 were published between 2004 and 2023, indicating these papers have been frequently cited over nearly two decades. Importantly, 15 of these papers are currently at their citation peak, suggesting that research on DMD will continue to attract attention in the future (**Supplementary Figure 4**).

In this field, among the 786 strongest emerging keywords, we focused on the 50 most impactful (**Supplementary Figure 5**). These keywords represent the current hotspots in the research area and likely future research directions.

## 4 Discussion

### 4.1 General information

This study employed bibliometric analysis to investigate publications related to DMD. The findings reveal the current state, hotspots, research trends, gaps for future studies, and contributions and collaborations among countries. Gaining a deep understanding of DMD-related research can provide direction for future research and practice. Such understanding is crucial for healthcare professionals to make more informed and effective decisions, ultimately improving patient health outcomes. Therefore, it has significant clinical implications. In today’s vast literature landscape, grasping an overview of the research field is particularly urgent, and bibliometrics can meet this need.

This paper is the first to combine bibliometric methods with content analysis to conduct mathematical statistical analysis of DMD-related literature and to identify research hotspots and trends based on data mining. Typically, four processes are required to obtain a standard CiteSpace knowledge map: software installation, retrieval and download of original articles, software function operation (including adjusting various parameters) and graphic interpretation. To collect articles accurately and comprehensively, authors should pay attention to data retrieval methods, choose appropriate parameters based on their needs and adjust the cluttered spectrum to obtain a clear and attractive spectrum.

An analysis of 9,277 DMD-related articles included in this study from 2004 to 2023 showed that 9,277 documents on DMD are present in the WoSCC database. Since 2004, the number of papers published annually has slowly increased. The research was divided into three phases: slow growth from 2004 to 2007 with fewer than 300 papers published annually, indicating little attention in the field; gradual increase from 2008 to 2013 as the field attracted researchers’ attention; and a rapid increase in publications after 2014 with a peak in 2021, indicating widespread attention in the field post-2014. Research on DMD applications has been conducted in 104 countries and regions. We predict that more studies that provide in-depth knowledge and insightful understanding of DMD will be published subsequently.

### 4.2 Knowledge base

This study employed bibliometric methods to analyze the literature on DMD. Results elucidate the current state, hotspots and trends in DMD research, as well as gaps for future study and international contributions and collaborations. A thorough understanding of DMD-related research is essential to guide future work and inform clinical practices, enhancing healthcare outcomes. Therefore, it holds significant clinical relevance. Given the vast amount of literature available, an overview of the field is crucial, and bibliometrics fulfils this requirement.

This article represents the first instance of integrating bibliometric methods with content analysis to perform mathematical statistical analysis on DMD-related literature, identifying research hotspots and trends through data mining. Typically, to produce a standard CiteSpace knowledge map, four steps are necessary: software installation, retrieval and download of original articles, operation of software features (including adjustment of various parameters) and graphic interpretation. To ensure accurate and comprehensive article collection, authors must carefully consider their data retrieval strategies and adjust parameters accordingly to clarify and improve the spectra.

An analysis of 9,277 DMD-related articles from 2004 to 2023 included in this study found that the WoSCC database contains 9,277 documents on DMD. Since 2004, published papers have increased gradually every year. The publication timeline is segmented into three phases: slow growth from 2004 to 2007 with fewer than 300 papers annually, indicating an initial lack of focus; increased volume from 2008 to 2013 as the field gained visibility; and a rapid rise post-2014 with a peak in 2021, reflecting broad interest in the field. Research on DMD applications has been conducted across 104 countries and regions. It is anticipated that more comprehensive and profound research on DMD will emerge and be published subsequently.

The impact of journals is determined by the frequency of their co-citation. *Neuromuscular Disorders* is the most co-cited journal with 5,769 citations, followed by *Muscle & Nerve* with 4,892 citations, and *Proceedings of the National Academy of Sciences of the United States of America* with 4,282 citations. Among the top 10 most co-cited journals, *Nature* has been cited 3,971 times and possesses the highest IF (64.8), indicating its significant influence on DMD research. Moreover, 80% of the frequently co-cited journals are in the Q1/Q2 categories. This study considers both the quantity and quality of journals, identifying the core journals in DMD research. Authors interested in this field should pay attention to these journals and consider them when preparing their manuscripts.

Among all authors who have published on DMD, the top 10 authors have collectively published 913 papers, accounting for 9.84% of all papers in this field. CiteSpace was used to visualize the network among these authors. The co-citation network, spanning 2004 to 2023, includes 2,060 nodes and 11,338 links. The most cited article is ‘Diagnosis and management of Duchenne muscular dystrophy, part 1: diagnosis, and neuromuscular, rehabilitation, endocrine, and gastrointestinal and nutritional management’ (24). This paper updates the 2010 DMD care considerations to improve patient care and address the needs of patients with extended survival times, and incorporates the implications of emerging DMD genetic and molecular therapies. It introduces three new topics: primary care and emergency management, endocrine management and care transitions throughout patients’ life span.

The second most cited article is ‘Diagnosis and management of Duchenne muscular dystrophy, part 2: diagnosis, and pharmacological and psychosocial management’. It provides updated recommendations for managing respiratory and cardiac health, osteoporosis, and orthopedic and surgical treatments for boys and men with DMD (25). It also offers guidance for cardiac treatment in female carriers of the mutation. The updated care considerations advocate the use of glucocorticoids to influence the natural progression of DMD and emphasize the need for lifelong care guidance as patient life spans extend. With the advent of new genetic and molecular therapies, significant changes in the treatment approaches for DMD are anticipated.

The third most cited article is titled ‘Diagnosis and management of Duchenne muscular dystrophy, part 1: diagnosis, and pharmacological and psychosocial management’ (26). This article establishes a framework for identifying the primary manifestations and secondary complications of DMD. It presents a method for formulating recommendations and offers a holistic view of nursing, pharmacological treatments and psychosocial management. The aforementioned three articles collectively underscore that treatment for DMD should adopt a multidisciplinary approach, highlighting the significant role of nursing within the therapeutic spectrum.

The above three articles further illustrate that the treatment of DMD is moving towards a multidisciplinary approach (27–32). These publications primarily discuss various treatment methods for DMD, including systemic and local drug therapies such as PRO051, phosphorodiamidate morpholino oligomer and corticosteroid treatments, as well as applications of gene editing technologies. The studies explore the long-term effects of these treatments on improving muscle function, quality of life and survival rates. The common goal of these studies is to restore or enhance muscle function in patients through different therapeutic strategies, thereby delaying the progression of DMD. These articles are also fundamental literature in the field of DMD (33–40).

### 4.3 Analysis of hotspots and emerging topics

We conducted co-citation reference clustering and temporal clustering analysis and discovered that early research hotspots include muscular dystrophy (cluster 3), transplantation (cluster 7), MLPA (cluster 10) and zonula occludens (cluster 13) (41–45). Mid-term research focuses were exon skipping (cluster 5), oxidative stress (cluster 6), utrophin (cluster 8) and satellite cells (cluster 9) (37, 46–50). Recent and trending topics in the field are DMD (cluster 0), CRISPR (cluster 1), eteplirsen (cluster 2), mitochondria (cluster 4), MRI (cluster 11), cognition (cluster 12) and structural variants (cluster 14) (1, 51–56).

By analyzing keywords, we can understand the status and direction of development in a field. On the basis of keyword co-occurrences in VOSviewer, the most popular keyword is ‘inflammation’ (269 occurrences), followed by ‘expression’ (207), ‘oxidative stress’ (143) and ‘activation’ (137).

We removed non-relevant keywords and constructed a network containing 180 keywords that appeared at least 12 times, resulting in five distinct clusters. Cluster 1 (red) contains 53 keywords including ‘skeletal-muscle’, ‘mdx’, ‘fibrosis’, ‘injury’, ‘differentiation’, ‘mice’, ‘stem-cell’, ‘self-renewal’, ‘regeneration’, ‘activation’, ‘damage’, ‘inflammation’, ‘proliferation’, ‘myogenesis’, ‘mechanisms’, ‘mitochondria’, ‘exercise’, ‘metabolism’, ‘growth’, ‘calcium’, and ‘animal models’. These terms focus on the structure and function of skeletal muscle and its repair processes after injury, specifically in the study of DMD, where mdx mice serve as a crucial animal model. These keywords describe the initiation of inflammation following muscle damage, the activation of muscle stem cells (such as satellite cells), self-renewal, differentiation and muscle regeneration processes, as well as the key roles of mitochondria, metabolism and calcium signaling in muscle maintenance and repair. Understanding these mechanisms is vital for developing therapeutic strategies to alleviate or repair muscle damage (43–45, 47, 57).

Cluster 2 (green) comprises 51 keywords including ‘children’, ‘boys’, ‘management’, ‘disease’, ‘atrophy’, ‘double-blind’, ‘disorders’, ‘prevalence’, ‘adolescents’, ‘scoliosis’, ‘disability’, ‘health’, reliability, ‘neuromuscular disease’, ‘trial’, ‘deflazacort’, ‘natural history’ and pulmonary ‘function’. These keywords primarily focus on the clinical management and research of neuromuscular diseases, especially concerning how these conditions are managed in children and adolescents. The vocabulary covers epidemiology of diseases, muscle atrophy, respiratory function impairments and disability issues related to conditions such as scoliosis. The terms also mention clinical trials using specific medications, such as deflazacort, particularly those employing double-blind methodologies to evaluate the effectiveness and reliability of treatments. Overall, these keywords describe systematic studies and management strategies aimed at improving the health and quality of life of affected children and adolescents (36, 58–60).

Cluster 3 (blue) contains 34 keywords, including ‘gene’, ‘duchenne’, ‘dmd’, ‘dystrophin’, ‘protein’, ‘mutation’, ‘carriers’, ‘DNA’, ‘deletion’, ‘locus’, ‘duplications’, ‘phenotype’, ‘complex’, ‘cancer’, ‘deficiency’, ‘molecular basis’, ‘central nervous system’, ‘database’ and ‘association’. These keywords indicate that mutations in DMD may involve deletions or duplications affecting specific genetic loci. Carriers often do not exhibit symptoms but can pass the mutant gene to their offspring. The phenotype of DMD is complex, with severity ranging from muscle weakness to impacts on the central nervous system. Currently, specialized databases have been established for the molecular basis of DMD, its genetic associations and disease management. Some relevance to cancer research may also exist (61–65).

Cluster 4 (yellow) includes 27 keywords, such as ‘expression’, ‘mdx mouse’, ‘utrophin’, ‘gene therapy’, ‘delivery’, ‘messenger RNA’, ‘restoration’, ‘systemic delivery’, ‘exon skipping’, ‘skeletal’, ‘canine model’, ‘pathology’ and ‘efficacy’. These keywords involve research in the field of gene therapy for muscular dystrophies. They primarily include using mdx mice and canine models to study systemic mRNA delivery and exon skipping techniques to restore skeletal muscle function impaired by the absence of utrophin protein. The goal of this research is to explore and evaluate the potential of these therapeutic strategies to improve muscle pathology and enhance efficacy (34, 66–68).

Cluster 5 (purple) comprises 18 keywords, including ‘biomarker’, ‘age’, ‘disease progression’, ‘dysfunction’, ‘heart’, ‘heart failure’, ‘MRI’, ‘involvement’, ‘myopathy’ and ‘tissue’. These keywords focus on using biomarkers to study how cardiac functions degrade with age and how this degradation leads to heart failure. Imaging techniques such as MRI allow for observation of the involvement of the heart and other muscle tissues, such as in myopathies, to understand the specific mechanisms of disease progression. This research area emphasizes identifying and evaluating biomarkers of heart disease and related muscle dysfunctions at different stages, providing a basis for diagnosis and treatment (13, 35, 69, 70).

We utilized CiteSpace to generate a volcano plot, which clearly illustrates the shifts in research hotspots over time through co-cited references and keywords. In this analysis, we identified 50 of the most robust citation bursts in DMD research. The reference with the highest burst strength (148.96) was published in *Lancet Neurology*, titled ‘Diagnosis and management of Duchenne muscular dystrophy, part 1: diagnosis, and neuromuscular, rehabilitation, endocrine, and gastrointestinal and nutritional management’ by Birnkrant (25). This article updates the 2010 DMD care considerations, adding three new topics: primary care and emergency management, endocrine management and care transitions throughout the life span. It introduces new guidelines on the diagnosis and management of DMD, addressing neuromuscular, rehabilitation, endocrine and gastrointestinal aspects. Key topics include care for female carriers of DMD, new molecular and gene therapy strategies, advanced rehabilitation assessments and technologically supported treatment approaches. Furthermore, it covers new guidelines on endocrine issues, management of DMD-specific nutritional problems, the potential of non-invasive prenatal testing and personalized treatment plans based on specific genetic features of the disease. The discussion also addresses the optimal timing for treatment interventions, the effects of long-term glucocorticoid therapy and how advancements in technology such as robotic aids can enhance the quality of life for patients. In addition, the article underscores the importance of gastrointestinal and nutritional management, including studies on the energy needs of DMD patients and the development of specific nutritional strategies (25–27).

In this study, we focused on the 50 keywords with the strongest bursts among 786 prominent emerging keywords in the field, primarily involving areas of biomedical research, genetics, and clinical trials. These terms included ‘muscle stem cells’, ‘rare diseases’, ‘gene editing’, ‘biomarkers’ and ‘rehabilitation’. These keywords reflect the pursuit of a deeper understanding of disease mechanisms and scientific efforts to intervene and manage diseases through genetic manipulation and effective treatments (38, 71–74).

These keywords represent the current research hotspots and indicate potential future directions in the field. Among the 50 referenced publications, 47 were published between 2004 and 2023, indicating their frequent citation over nearly two decades. Notably, 15 of these papers are currently at their citation peak, suggesting that research on DMD will continue to be a focal point in the future.

### 4.4 Limitations

This study has certain limitations. Firstly, it focuses solely on publications from the WoSCC database and does not include other databases such as PubMed and Scopus, which might yield slightly different results. Although WoSCC is a comprehensive and popular online database in scientometrics, several papers on this topic may have been published in journals that are not indexed by this network. Secondly, non-English publications were excluded. Thus, milestone articles published in other languages were not considered, leading to the omission of potentially significant works. Lastly, citation counts do not fully reflect the quality of a paper because citing articles takes time. Older journals may receive more citations, and influential manuscripts might take years to accumulate citations.

## 5 Conclusions

To our knowledge, this study represents the first bibliometric analysis of publications in the DMD research field using visualization software and data mining techniques, providing insights into the current status, hotspots and developmental trends of the field. Research in this area predominantly concentrates on DMD-related studies, with potential future research hotspots likely focusing on precision medicine for DMD. The mechanisms involved need to be explored further to provide a theoretical basis for clinical application. Precision medicine in DMD seeks to tailor treatments based on the individual genetic, environmental and lifestyle factors of each patient. The advent of next-generation sequencing and advanced genomic technologies has enabled the identification of specific mutations within the DMD gene, facilitating more personalized therapeutic approaches. For instance, exon skipping therapies designed to bypass faulty gene segments have shown promise in clinical trials for certain mutations. In addition, gene therapy strategies, including CRISPR-Cas9 mediated gene editing and microdystrophin replacement, are being investigated to correct or compensate for the defective gene.

Despite these advancements, several challenges remain in translating these findings into clinical practice. The heterogeneity of mutations and the complex pathophysiology of DMD necessitate a deeper understanding of the underlying mechanisms driving the disease. Research focusing on molecular and cellular pathways affected by dystrophin deficiency, such as inflammation, fibrosis and muscle regeneration, is crucial for developing effective treatments. Moreover, optimizing delivery methods to ensure these therapies reach the affected tissues efficiently and safely is essential. Integrating precision medicine into routine clinical care also requires a robust infrastructure and collaboration among multidisciplinary teams. Clinicians, geneticists and researchers must work together to interpret genetic data accurately and develop individualized treatment plans. Establishing comprehensive patient registries and biobanks can facilitate this process by providing valuable data for ongoing research and therapy development. In conclusion, while precision medicine holds significant potential for improving DMD management, addressing scientific, technical and clinical challenges is also imperative. Continued research into the disease mechanisms and the development of innovative therapeutic approaches will be pivotal in making precision medicine a reality for DMD patients.

DMD researchers and practitioners can utilize the findings from this study to enhance their understanding of the field and foster further knowledge development. The results can also inform novice researchers, interested readers, or research managers and evaluators who lack specific knowledge, aiding in their development of perspectives on DMD. The outputs of this study can serve as a guide for further research and as a starting point for more formal syntheses of knowledge, such as systematic reviews and meta-analyses. This comprehensive approach helps bridge gaps in the existing literature and propels scientific and clinical understanding of DMD.

## Data Availability

The original contributions presented in the study are included in the article/**Supplementary Material**. Further inquiries can be directed to the corresponding author.

## Supplementary material

Supplementary Figure 1 Keyword Density Map. (Created using CiteSpace version: 6.1.6R, on January 17, 2024. Also utilized VOSviewer version: 1.6.17.).

Supplementary Figure 2 Keyword Clustering Volcano Plot (Created using CiteSpace version: 6.1.6R on January 17, 2024. Also utilized VOSviewer version: 1.6.17.).

Supplementary Figure 3 Keyword Clustering Diagram (Created using CiteSpace version: 6.1.6R on January 17, 2024. Also utilized VOSviewer version: 1.6.17.).

Supplementary Figure 4 Citation Burst Map (Created using CiteSpace version: 6.1.6R on January 17, 2024. Also utilized VOSviewer version: 1.6.17.).

Supplementary Figure 5 Keyword Burst Map (Created using CiteSpace version: 6.1.6R on January 17, 2024. Also utilized VOSviewer version: 1.6.17.).
