# Supplementary material for: Research hotspots and trends for Duchenne muscular dystrophy: a machine learning bibliometric analysis from 2004 to 2023
Source: Front Immunol. 2024 Nov 28;15:1429609. doi: 10.3389/fimmu.2024.1429609 (PMC11634759; doi:10.3389/fimmu.2024.1429609)
Supplement: Supplementary file 1 [file Table1.docx]

**Supplementary Tables**

**Table 1.** National Publication Volumes.

| Rank | Country/region | Article counts | Centrality | Percentage (%) | Citation | Citation per publication |
| --- | --- | --- | --- | --- | --- | --- |
| 1 | USA | 3524 | 0.27 | 37.99 | 153524 | 43.57 |
| 2 | ENGLAND | 1120 | 0.13 | 12.07 | 45035 | 40.21 |
| 3 | ITALY | 966 | 0.11 | 10.41 | 35565 | 36.82 |
| 4 | FRANCE | 844 | 0.19 | 9.10 | 30436 | 36.06 |
| 5 | CANADA | 669 | 0.07 | 7.21 | 26829 | 40.10 |
| 6 | JAPAN | 655 | 0.03 | 7.06 | 18566 | 28.35 |
| 7 | CHINA | 592 | 0.07 | 6.38 | 10336 | 17.46 |
| 8 | NETHERLANDS | 516 | 0.18 | 5.56 | 19850 | 38.47 |
| 9 | GERMANY | 495 | 0.06 | 5.34 | 17473 | 35.30 |
| 10 | AUSTRALIA | 474 | 0.08 | 5.11 | 18329 | 38.67 |

**Table 2.** Institutional Publications.

| Rank | Institution | Country | Number of studies | Total citations | Average citation |
| --- | --- | --- | --- | --- | --- |
| 1 | Institut National de la Sante et de la Recherche Medicale (Inserm) | France | 483 | 17546 | 36.33 |
| 2 | University of London | England | 451 | 18002 | 39.92 |
| 3 | University of California System | USA | 423 | 23662 | 55.94 |
| 4 | University System of Ohio | USA | 391 | 18982 | 48.55 |
| 5 | Centre National de la Recherche Scientifique (CNRS) | France | 353 | 14846 | 42.06 |
| 6 | University College London | England | 346 | 14366 | 41.52 |
| 7 | Sorbonne Universite | France | 333 | 13925 | 41.82 |
| 8 | Ohio State University | USA | 306 | 16630 | 54.35 |
| 9 | Leiden University | Netherlands | 290 | 12301 | 42.42 |
| 10 | Leiden University Medical Center (LUMC) | Netherlands | 287 | 12210 | 42.54 |

**Table 3.** Journal Publication Volume.

| Rank | Journal | Article counts | Percentage (9277) | IF | Quartile in category |
| --- | --- | --- | --- | --- | --- |
| 1 | *Neuromuscular Disorders* | 396 | 4.27 | 2.8 | Q3 |
| 2 | *Muscle & Nerve* | 326 | 3.51 | 3.4 | Q2 |
| 3 | *Plos One* | 298 | 3.21 | 3.7 | Q2 |
| 4 | *Human Molecular Genetics* | 182 | 1.96 | 3.5 | Q3 |
| 5 | *Molecular Therapy* | 152 | 1.64 | 12.4 | Q1 |
| 6 | *International Journal of Molecular Sciences* | 141 | 1.52 | 5.6 | Q1 |
| 7 | *Scientific Reports* | 119 | 1.28 | 4.6 | Q2 |
| 8 | *Journal of Neuromuscular Diseases* | 97 | 1.05 | 3.3 | Q2 |
| 9 | *Human Gene Therapy* | 83 | 0.89 | 4.2 | Q2 |
| 10 | *Skeletal Muscle* | 81 | 0.87 | 4.9 | Q2 |

**Table 4.** Journal Co-citation.

| Rank | Cited Journal | Co-Citation | IF (2020) | Quartile in category |
| --- | --- | --- | --- | --- |
| 1 | NEUROMUSCULAR DISORD | 5769 | 2.8 | Q3 |
| 2 | MUSCLE NERVE | 4892 | 3.4 | Q2 |
| 3 | P NATL ACAD SCI USA | 4282 | 11.1 | Q1 |
| 4 | CELL | 3997 | 64.5 | Q1 |
| 5 | NATURE | 3971 | 64.8 | Q1 |
| 6 | HUM MOL GENET | 3757 | 3.5 | Q3 |
| 7 | PLOS ONE | 3685 | 3.7 | Q2 |
| 8 | NEUROLOGY | 3329 | 10.1 | Q1 |
| 9 | J BIOL CHEM | 2820 | 4.8 | Q2 |
| 10 | SCIENCE | 2805 | 56.9 | Q1 |

**Table 5.** Author Publications and Co-citations.

| Rank | Author | Count | Location | Rank | Co-cited author | Citation |
| --- | --- | --- | --- | --- | --- | --- |
| 1 | Muntoni, Francesco | 140 | USA | 1 | HOFFMAN EP | 2099 |
| 2 | Aartsma-Rus, Annemieke | 118 | Netherlands | 2 | MENDELL JR | 1531 |
| 3 | Takeda, Shin’ichi | 99 | USA | 3 | BUSHBY K | 1402 |
| 4 | Duan, Dongsheng | 96 | China | 4 | AARTSMA-RUS A | 1208 |
| 5 | Hoffman, Eric P. | 84 | USA | 5 | KOENIG M | 1038 |
| 6 | Straub, Volker | 84 | USA | 6 | MUNTONI F | 1035 |
| 7 | McDonald, Craig M. | 76 | USA | 7 | MCDONALD CM | 989 |
| 8 | Chamberlain, Jeffrey S. | 72 | USA | 8 | EMERY AEH | 931 |
| 9 | Flanigan, Kevin M. | 72 | USA | 9 | BIRNKRANT DJ | 853 |
| 10 | Mendell, Jerry R. | 72 | USA | 10 | ERVASTI JM | 764 |

**Table 6.** Co-cited References.

| Rank | Title | Journal IF (2021) | Author(s) | Total citations |
| --- | --- | --- | --- | --- |
| 1 | Diagnosis and management of Duchenne muscular dystrophy, part 1: diagnosis, and neuromuscular, rehabilitation, endocrine, and gastrointestinal and nutritional management | *LANCET NEUROLOGY (IF=48.00)* | Birnkrant DJ | 489 |
| 2 | Diagnosis and management of Duchenne muscular dystrophy, part 2: respiratory, cardiac, bone health, and orthopaedic management | *LANCET NEUROLOGY (IF=48.00)* | Birnkrant DJ | 356 |
| 3 | Diagnosis and management of Duchenne muscular dystrophy, part 1: diagnosis, and pharmacological and psychosocial management | *LANCET NEUROLOGY (IF=48.00)* | Bushby K | 307 |
| 4 | Systemic Administration of PRO051 in Duchenne’s Muscular Dystrophy | *NEW ENGLAND JOURNAL OF MEDICINE (IF=158.5)* | Goemans NM | 263 |
| 5 | Exon skipping and dystrophin restoration in patients with Duchenne muscular dystrophy after systemic phosphorodiamidate morpholino oligomer treatment: an open-label, phase 2, dose-escalation study | *LANCET (IF=168.9)* | Cirak S | 254 |
| 6 | Long-term effects of glucocorticoids on function, quality of life, and survival in patients with Duchenne muscular dystrophy: a prospective cohort study | *LANCET (IF=168.9)* | McDonald CM | 211 |
| 7 | Duchenne muscular dystrophy | *NATURE REVIEWS DISEASE PRIMERS (IF=81.5)* | Duan DS | 209 |
| 8 | Local restoration of dystrophin expression with the morpholino oligomer AVI-4658 in Duchenne muscular dystrophy: a single-blind, placebo-controlled, dose-escalation, proof-of-concept study | *LANCET NEUROLOGY (IF=48.00)* | Kinali M | 195 |
| 9 | Local dystrophin restoration with antisense oligonucleotide PRO051 | *NEW ENGLAND JOURNAL OF MEDICINE (IF=158.5)* | van Deutekom JC | 194 |
| 10 | In vivo genome editing improves muscle function in a mouse model of Duchenne muscular dystrophy | *SCIENCE* (IF=56.9) | Nelson CE | 179 |

**Table 7.**High-Frequency Keywords.

| Rank | Keyword | Counts | Rank | Keyword | Counts |
| --- | --- | --- | --- | --- | --- |
| 1 | skeletal-muscle | 1571 | 11 | therapy | 439 |
| 2 | expression | 1493 | 12 | diagnosis | 392 |
| 3 | children | 771 | 13 | satellite cells | 380 |
| 4 | dystrophin | 761 | 14 | mutations | 373 |
| 5 | gene | 725 | 15 | management | 372 |
| 6 | duchenne | 651 | 16 | skeletal muscle | 372 |
| 7 | mice | 629 | 17 | glycoprotein complex | 368 |
| 8 | mdx mice | 572 | 18 | protein | 365 |
| 9 | mdx mouse | 508 | 19 | regeneration | 356 |
| 10 | dmd | 504 | 20 | boys | 348 |
