# Supplementary figures and images for: Research hotspots and trends for Duchenne muscular dystrophy: a machine learning bibliometric analysis from 2004 to 2023
Source: Front Immunol. 2024 Nov 28;15:1429609. doi: 10.3389/fimmu.2024.1429609 (PMC11634759; doi:10.3389/fimmu.2024.1429609)

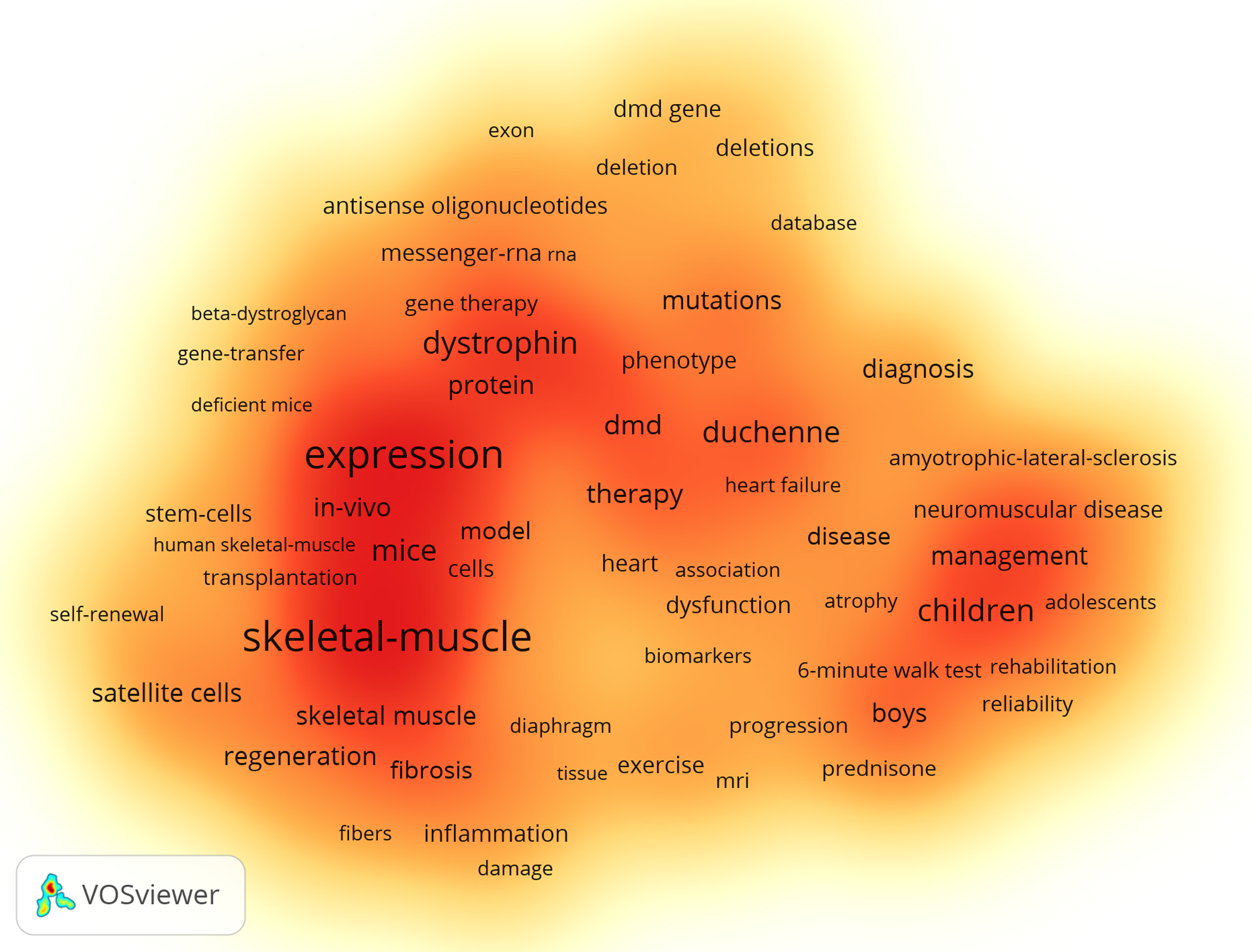

Supplement: Supplementary Figure 1 — Keyword Density Map. (Created using CiteSpace version: 6.1.6R, on January 17, 2024. Also utilized VOSviewer version: 1.6.17.). [file Image1.tif]

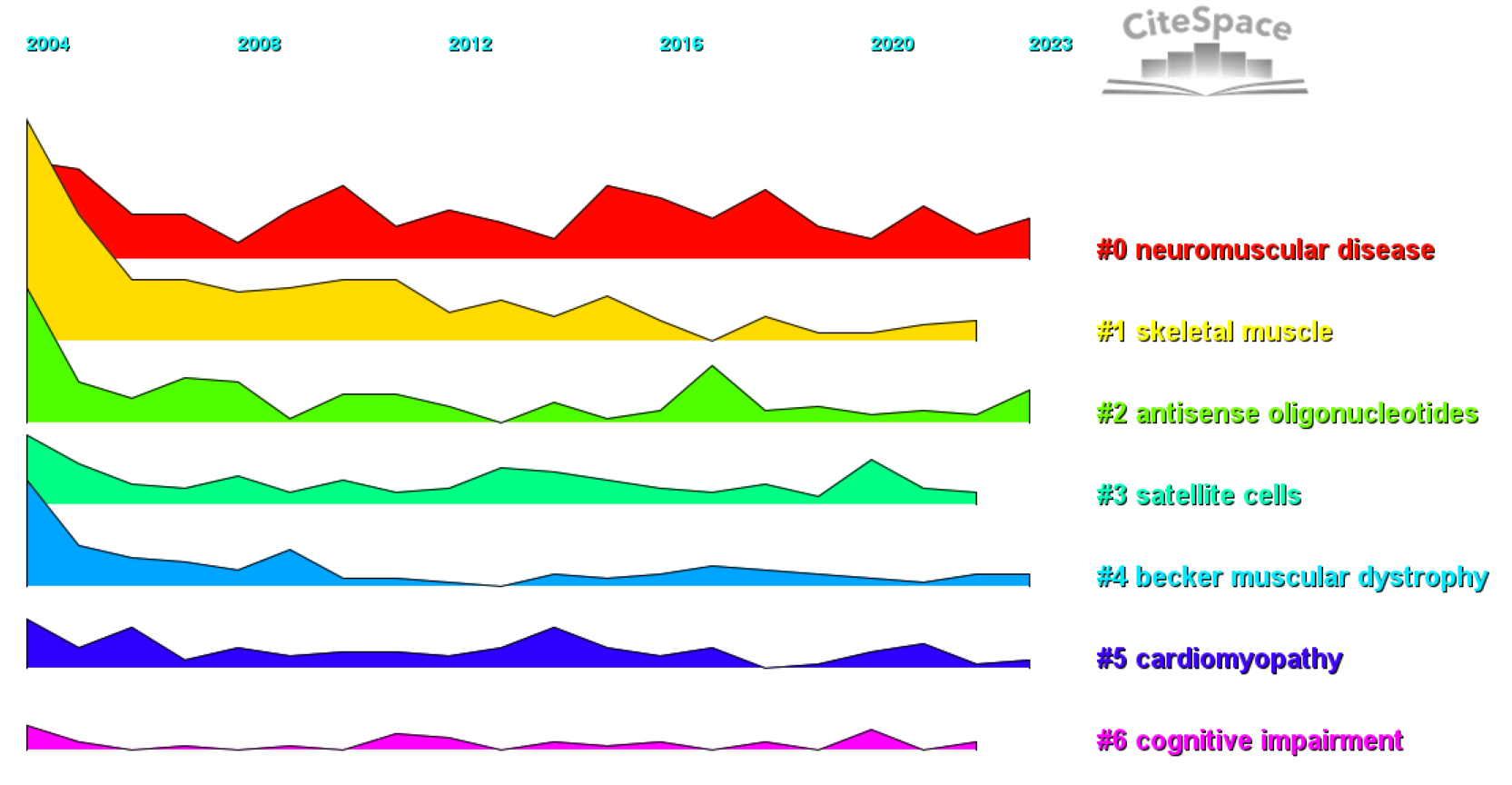

Supplement: Supplementary Figure 2 — Keyword Clustering Volcano Plot (Created using CiteSpace version: 6.1.6R on January 17, 2024. Also utilized VOSviewer version: 1.6.17.). [file Image2.tif]

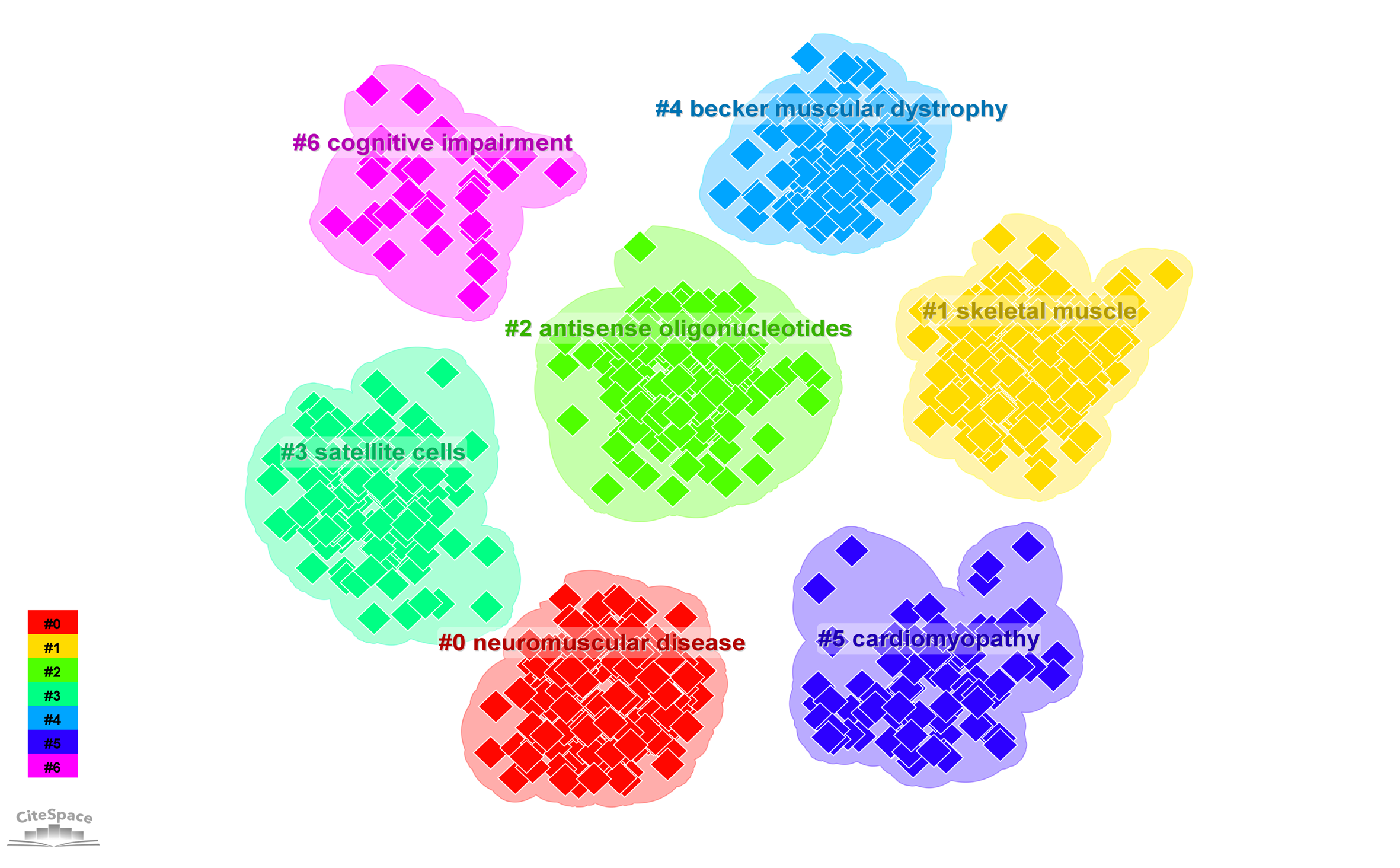

Supplement: Supplementary Figure 3 — Keyword Clustering Diagram (Created using CiteSpace version: 6.1.6R on January 17, 2024. Also utilized VOSviewer version: 1.6.17.). [file Image3.tif]

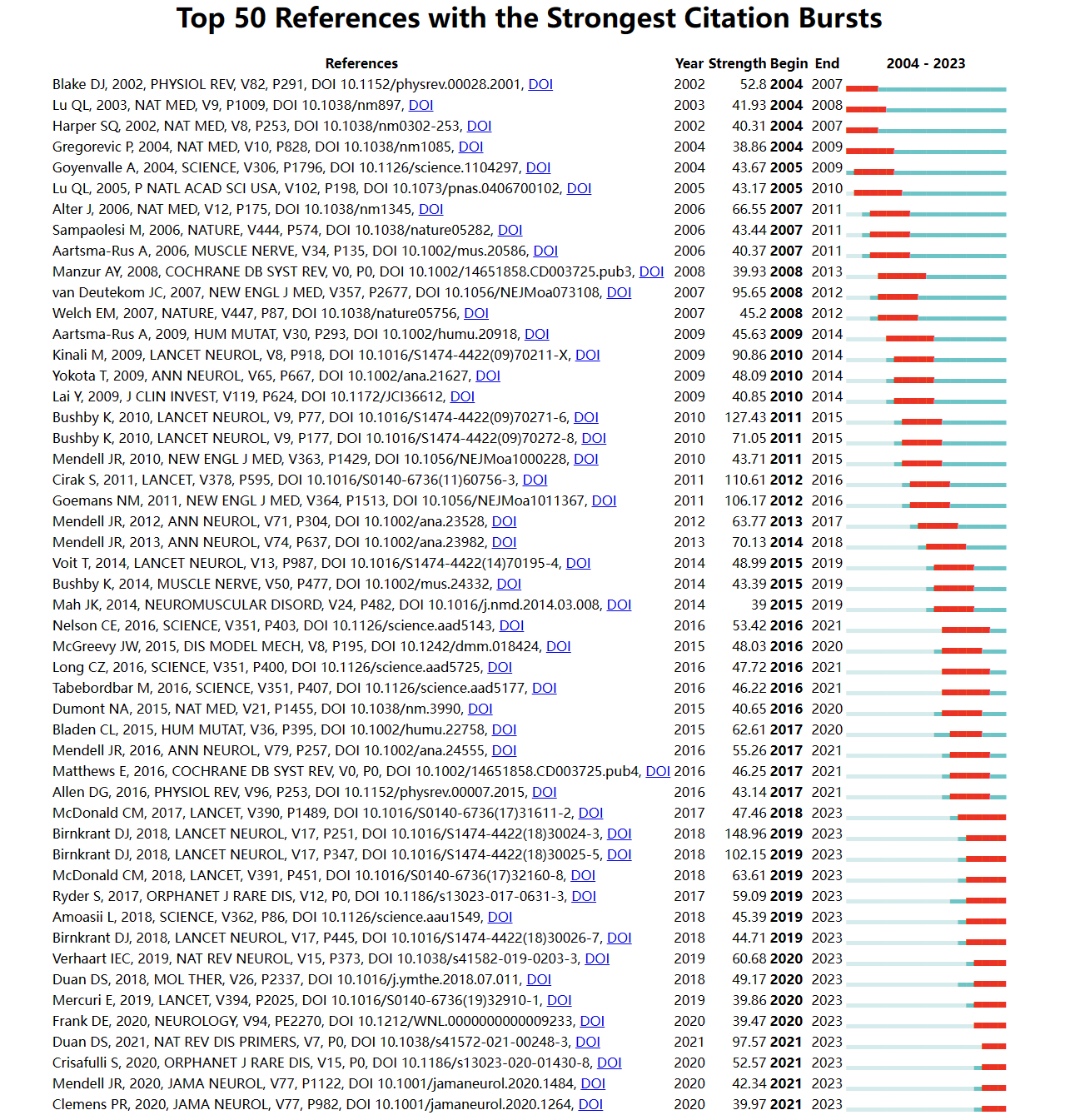

Supplement: Supplementary Figure 4 — Citation Burst Map (Created using CiteSpace version: 6.1.6R on January 17, 2024. Also utilized VOSviewer version: 1.6.17.). [file Image4.tif]

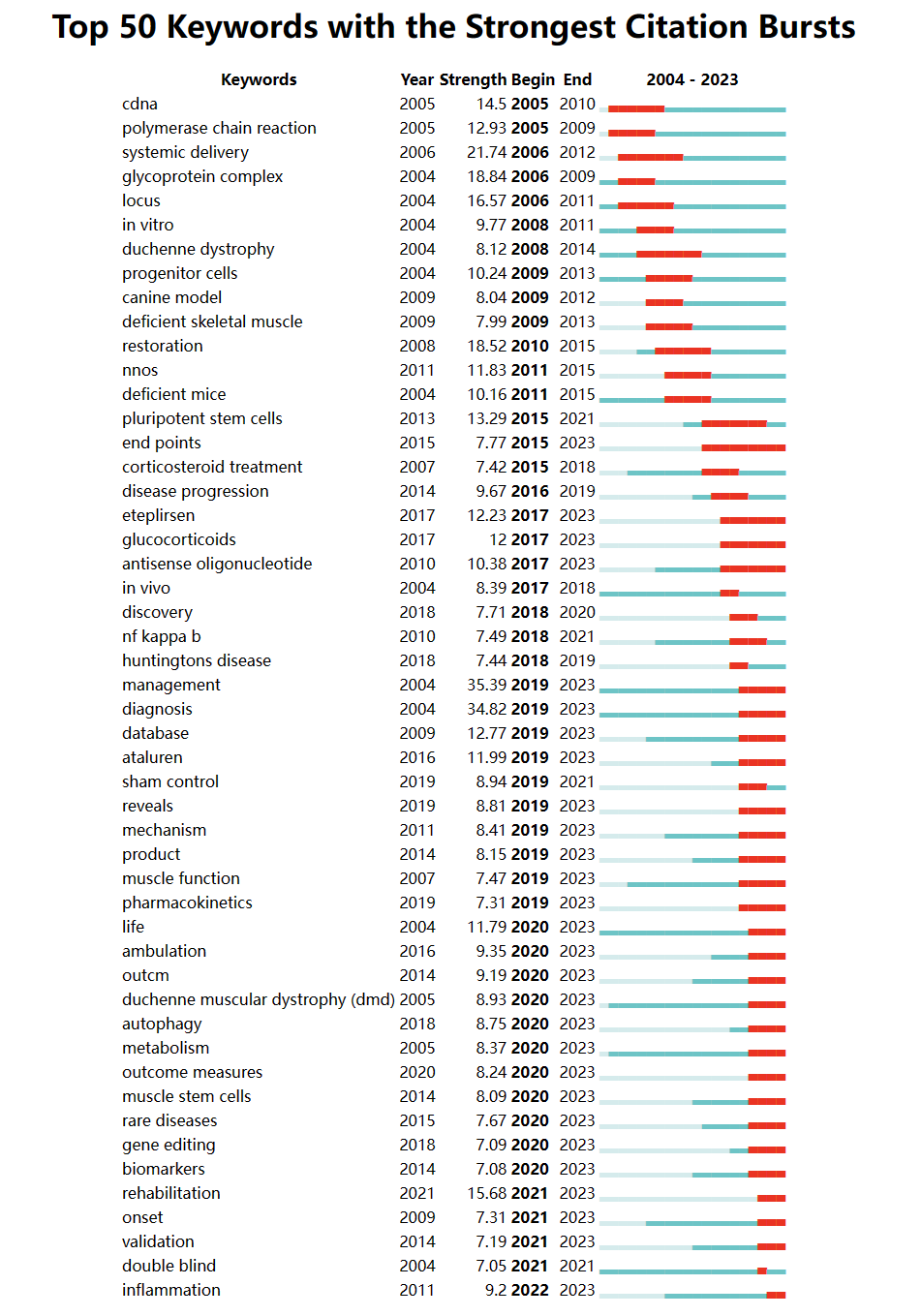

Supplement: Supplementary Figure 5 — Keyword Burst Map (Created using CiteSpace version: 6.1.6R on January 17, 2024. Also utilized VOSviewer version: 1.6.17.). [file Image5.tif]
